# Neonatal Colonization With Antibiotic-Resistant Pathogens in Low- and Middle-Income Countries: A Systematic Review and Meta-Analysis

**DOI:** 10.1001/jamanetworkopen.2024.41596

**Published:** 2024-11-05

**Authors:** Anne-Lise Beaumont, Elsa Kermorvant-Duchemin, Sébastien Breurec, Bich-Tram Huynh

**Affiliations:** 1 Anti-Infective Evasion and Pharmacoepidemiology Team, Center for Epidemiology and Population Health, Université Paris-Saclay, UVSQ, INSERM, Montigny-le-Bretonneux, France; 2 Epidemiology and Modelling of Antibiotic Evasion, Institut Pasteur, Université Paris Cité, Paris, France; 3 Department of Neonatal Medicine, AP-HP, Hôpital Necker-Enfants Malades, Université Paris Cité, Paris, France; 4 Clinical Microbiology Group, Institut Pasteur of Guadeloupe, Les Abymes, France; 5 Clinical Microbiology Laboratory, University Hospital of Guadeloupe, Pointe-à-Pitre, Guadeloupe, France; 6 Clinical Investigation Center Antilles–Guyane, Unit 1424, INSERM, Pointe-à-Pitre, Guadeloupe, France; 7 Pathogenesis and Control of Chronic and Emerging Infections, University of Montpellier, INSERM, Établissement français du sang, University of Antilles, Pointe-à-Pitre, France

## Abstract

**Importance:**

In low- and middle-income countries (LMICs), neonatal bacterial infections are mainly caused by Enterobacterales species and *Staphylococcus aureus*, which are also the leading causes of mortality directly attributable to antimicrobial resistance. As bacterial colonization often precedes infection, better knowledge of colonization is crucial to prevent antibiotic-resistant neonatal sepsis.

**Objective:**

To synthesize current evidence on the prevalence of and factors associated with colonization with third-generation cephalosporin–resistant Enterobacterales (3GCRE), carbapenem-resistant Enterobacterales (CRE), and methicillin-resistant *S aureus* (MRSA) during the first 3 months of life in LMICs.

**Data Sources:**

PubMed, Scopus, Web of Science, and the World Health Organization Global Index Medicus were searched for articles published from January 1, 2000, through July 29, 2024.

**Study Selection:**

Included studies were conducted in LMICs and reported prevalence rates or factors associated with colonization with 3GCRE, CRE, or MRSA in neonates and infants up to 3 months of age. Outbreak reports were excluded.

**Data Extraction and Synthesis:**

Data extraction and risk-of-bias assessment using a Joanna Briggs Institute tool were performed by 2 independent reviewers. Pooled prevalence for each pathogen was computed using a random-effects model. Reporting followed the Preferred Reporting Items for Systematic Reviews and Meta-Analyses guideline.

**Main Outcomes and Measures:**

Prevalence of and factors associated with 3GCRE, CRE, and MRSA colonization.

**Results:**

Of the 3147 articles identified in the search, 67 studies (51 for 3GCRE and CRE and 16 for MRSA) including 17 152 individuals were eligible. The pooled prevalence of 3GCRE colonization was 30.2% (95% CI, 21.4%-40.7%; τ^2^ = 1.48; *I*^2^ = 95.1%), varying from 18.2% (95% CI, 10.8%-29.1%) in nonhospitalized individuals to 48.2% (95% CI, 36.4%-60.2%) in hospitalized individuals. The prevalence of CRE colonization was 2.6% (95% CI, 0.7%-8.8%; τ^2^ = 7.79; *I*^2^ = 95.6%), while it was 2.7% (95% CI, 1.0%-6.7%; τ^2^ = 2.58; *I*^2^ = 93.5%) for MRSA. Increased risk of colonization with 3GCRE was associated with hospital birth (odds ratio [OR], 1.87; 95% CI, 1.33-2.64), neonatal antibiotic use (OR, 2.96; 95% CI, 1.43-6.11), and prolonged rupture of membranes (OR, 3.86; 95% CI, 2.19-6.84).

**Conclusions and Relevance:**

In this systematic review and meta-analysis of antibiotic-resistant pathogen carriage in individuals aged 0 to 3 months, the pooled prevalence was substantial despite a limited exposure period. Although high heterogeneity between studies limited extrapolation of results, the findings highlight the need for further investigation to identify transmission routes and to design targeted and effective preventive measures.

## Key Points

**Question:**

What are the prevalence of and factors associated with colonization with third-generation cephalosporin–resistant Enterobacterales (3GCRE), carbapenem-resistant Enterobacterales (CRE), and methicillin-resistant *Staphylococcus aureus* (MRSA) during the first 3 months of life in low- and middle-income countries (LMICs)?

**Findings:**

In this systematic review and meta-analysis of 67 studies including 17 152 individuals, the pooled prevalence of 3GCRE colonization was 30% compared with 3% for CRE and 3% for MRSA colonization. Hospital birth, previous neonatal antibiotic use, and prolonged rupture of membranes were associated with increased risk of 3GCRE colonization.

**Meaning:**

The findings suggest substantial prevalence of antibiotic-resistant pathogen carriage in neonates and infants in LMICs.

## Teaser

This systematic review and meta-analysis investigates the prevalence of colonization with antibiotic-resistant bacterial pathogens in the first 3 months of life in low- and middle-income countries (LMICs).

## Introduction

In low- and middle-income countries (LMICs), neonatal deaths account for almost half^1^ of mortality among individuals younger than 5 years. Infectious causes are involved in more than half of these deaths,^2^ with bacterial infections being the leading etiology,^3^ particularly *Klebsiella pneumoniae*, *Escherichia coli*, and *Staphylococcus aureus*.^4,5^ These have also been identified as the 3 main pathogens responsible for deaths directly attributable to antimicrobial resistance (AMR) in 2019.^6^

Assessing the burden of AMR in bacterial infections in LMICs is challenging due to limited access to health care and inadequate medical laboratory facilities. Documentation of neonatal infections is further complicated by sample collection challenges and the low positivity rate of blood cultures. Therefore, relying solely on neonatal infection studies^7^ may not provide a complete understanding of the problem. Conversely, the measurement of antibiotic-resistant bacterial colonization is less dependent on access to health care. Samples are easier to collect, and sensitivity is higher.^8^ Additionally, colonization often precedes subsequent infection and contributes to the spread of AMR. Therefore, colonization data can fill existing knowledge gaps and enhance understanding of AMR spread.^8^ The objectives of this systematic review and meta-analysis were to investigate the prevalence of colonization in the first 3 months of life with third-generation cephalosporin–resistant Enterobacterales (3GCRE), carbapenem-resistant Enterobacterales (CRE), and methicillin-resistant *S aureus* (MRSA) in LMICs and to identify factors associated with increased risk.

## Methods

This study was conducted according to the Meta-Analysis of Observational Studies in Epidemiology (MOOSE) and Preferred Reporting Items for Systematic Reviews and Meta-Analyses (PRISMA) reporting guidelines. The protocol was published in PROSPERO (CRD42023408406).

### Literature Search and Screening Strategy

All articles published between January 1, 2000, and July 29, 2024, were searched in PubMed, Scopus, Web of Science, and the World Health Organization (WHO) Global Index Medicus. The search strategy (eAppendix 1 in Supplement 1, reviewed by a specialist librarian) was divided into 2 parts: one to identify studies reporting risk factors for or prevalence of 3GCRE and/or CRE colonization and another for MRSA colonization. This was supplemented by handsearching the reference lists of all eligible studies. Search terms are listed in eAppendix 1 in Supplement 1.

### Study Selection and Review Process

Cohort and cross-sectional studies were eligible if they (1) reported the prevalence of or risk factors for 3GCRE, CRE, or MRSA colonization; (2) included a population from birth to 3 months of age; and (3) were conducted in an LMIC (according to the World Bank classification during the study period). Exclusion criteria were outbreak reports, studies focusing on a subpopulation with a specific resistance enzyme, or sample size less than 10. Of note, studies were not excluded if they included a baseline cross-sectional survey. Studies focusing on specific Enterobacterales species (eg, *E coli*) and case-control studies were included in the descriptive review but excluded from the prevalence meta-analysis. Two investigators (A.-L.B., B.-T.H.) independently screened titles and abstracts to identify relevant studies. Then, they assessed full-text articles for eligibility, extracted data, and evaluated methodologic quality using a modified version of the Joanna Briggs Institute Critical Appraisal Checklist for Prevalence Studies^9^ tailored to the research question (eAppendix 2 and eTable 1 in Supplement 1). If data could not be extracted directly from the article, the authors were contacted up to 4 times. Any discrepancies were resolved by discussion with E.K.-D. and S.B.

### Data Extraction

Parameters such as study design, year, setting, sample site, and inclusion and exclusion criteria; prevalence of and laboratory methods and guidelines used to detect resistance; and bacterial species, resistance genes, and results of risk factor analysis were extracted. Substudies within the same publication were treated as separate studies when reporting results. For cohort studies, data were extracted from the initial collection to ensure comparability with cross-sectional studies.

### Statistical Analysis

Individual study prevalences were calculated by dividing the number of individuals colonized with the antibiotic-resistant bacteria of interest by the total number of individuals who had samples obtained, with 95% Agresti-Coull CIs.^10^ A random-effects model was used to pool prevalence after logit transformation using generalized linear mixed-effects models^11^ and applying the Knapp-Hartung adjustment.^12^ The heterogeneity was expressed by the τ^2^ statistic^13^ and the *I*^2^ statistic.^14^ Evidence of publication bias was assessed using the Peters regression test.^15^

Different sensitivity analyses were performed excluding (1) studies using only a molecular-based microbiologic approach and (2) studies with a high risk of bias. The studies were divided into 2 subgroups according to the neonate’s or infant’s status at the time of sample obtainment: hospitalized, including individuals who were hospitalized for more than 24 hours, and nonhospitalized, including individuals who were not hospitalized at the time of sample obtainment. The latter category included neonates who had samples obtained at birth in a hospital, patients who had samples obtained during a medical consultation or at hospital admission, and those from the community. Analyses were performed using RStudio, version 2023.09.0 + 463 (RStudio, PBC) using meta and metafor packages.^16^ Statistical methods used for multiple meta-regression and risk factor meta-analysis are detailed in eAppendix 2 in Supplement 1.

## Results

**Figure 1.**
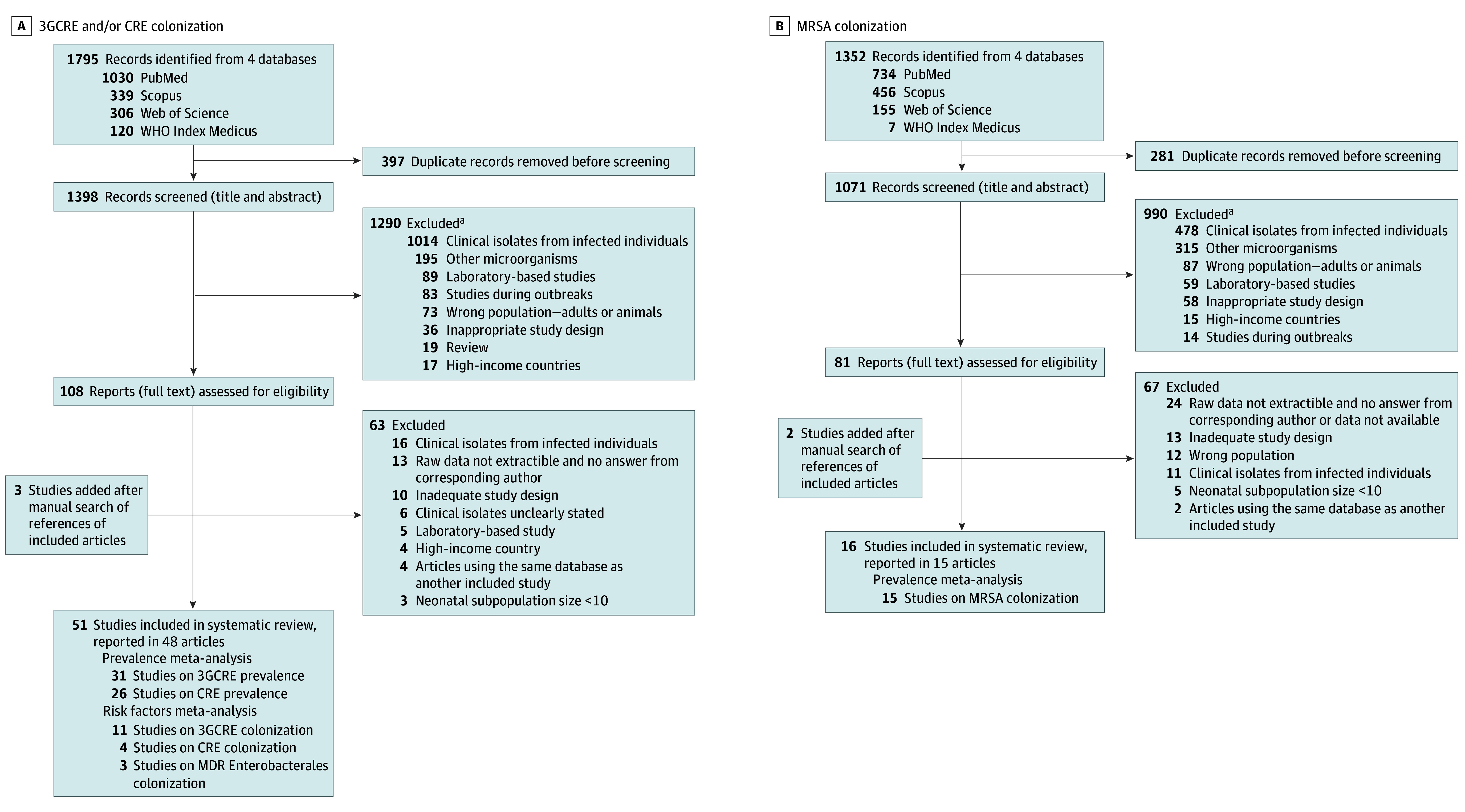
Flowchart of the Study Selection Process 3GCRE indicates third-generation cephalosporin–resistant Enterobacterales; CRE, carbapenem-resistant Enterobacterales; MDR, multidrug resistant; MRSA, methicillin-resistant *Staphylococcus aureus*; WHO, World Health Organization. ^a^Some studies were excluded for more than 1 reason.

Of the 3147 articles identified in our search, 67 studies including 17 152 individuals were eligible (Figure 1). Of these studies, 51 evaluated 3GCRE and CRE colonization^17,18,19,20,21,22,23,24,25,26,27,28,29,30,31,32,33,34,35,36,37,38,39,40,41,42,43,44,45,46,47,48,49,50,51,52,53,54,55,56,57,58,59,60,61,62,63,64^ and 16 evaluated MRSA colonization.^34,65,66,67,68,69,70,71,72,73,74,75,76,77,78^

### Colonization by 3GCRE and CRE

**Figure 2.**
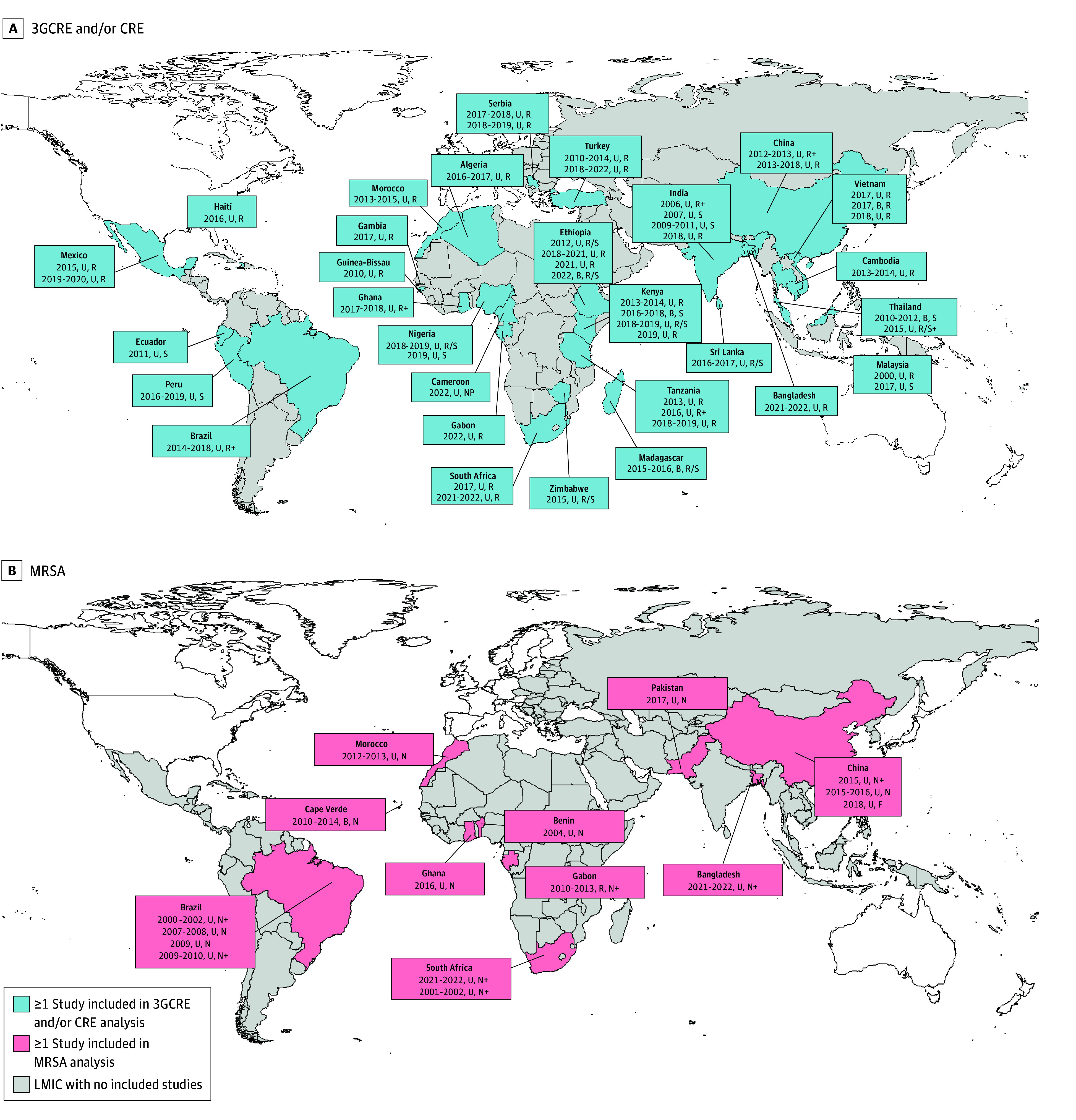
Geographic Distribution of Included Studies High-income countries are not shaded. Boxes indicate year of study realization, setting (B, both rural and urban; R, rural; U, urban), and sample type (F, fecal; N, nasal; NP, nasopharyngeal; R, rectal; S, stool; +, another sample site). 3GCRE indicates third-generation cephalosporin–resistant Enterobacterales; CRE, carbapenem-resistant Enterobacterales; LMIC, low- or middle-income country; MRSA, methicillin-resistant *Staphylococcus aureus*.

The database search yielded 1795 records pertaining to 3GCRE and CRE colonization (Figure 1), with 51 studies (11 572 individuals) being ultimately included (reported in 48 articles^17,18,19,20,21,22,23,24,25,26,27,28,29,30,31,32,33,34,35,36,37,38,39,40,41,42,43,44,45,46,47,48,49,50,51,52,53,54,55,56,57,58,59,60,61,62,63,64^). The most represented WHO region was Africa (24 studies [47.1%]; 3682 individuals [31.8%]).^18,20,22,24,26,27,30,31,32,33,34,36,37,44,45,46,47,48,49,57,58,60,62^ Almost half of the studies (24 [47.1%]) recruited hospitalized individuals,^17,23,28,30,31,34,37,38,47,48,49,51,52,53,54,55,56,57,58,60,63,64^ and nearly all of them (45 [88.2%]) were conducted in urban settings only^17,19,20,21,22,23,24,25,26,27,28,29,32,33,34,35,37,38,39,40,41,43,44,45,46,47,48,49,50,51,52,53,54,55,56,57,58,59,60,61,62,63,64^ (Figure 2). Sample obtainment and microbiologic techniques are detailed in eTables 2 and 3 and described in eAppendix 3 in Supplement 1.

**Figure 3.**
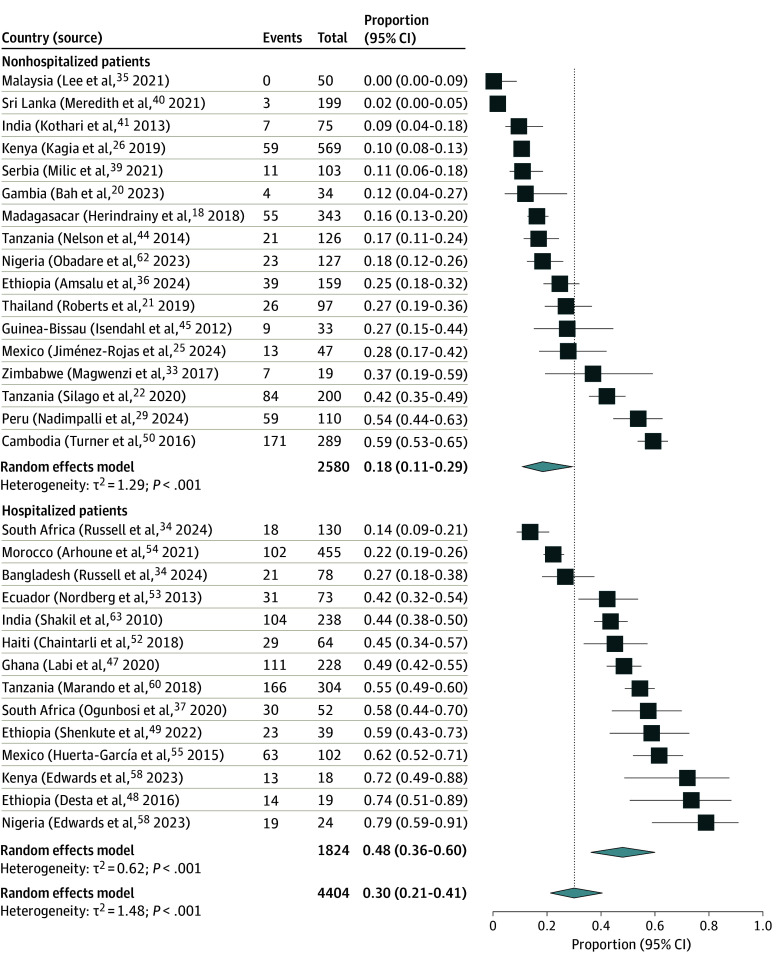
Third-Generation Cephalosporin–Resistant Enterobacterales Colonization Prevalence Stratified by Hospitalization Status

Based on 4404 individuals (38.1%) included in 31 studies (60.8%) (eTable 2 in Supplement 1), the pooled prevalence of 3GCRE colonization was 30.2% (95% CI, 21.4%-40.7%; τ^2^ = 1.48; *I*^2^ = 95.1%).^18,20,21,22,25,26,29,33,34,35,36,37,39,40,41,44,45,47,48,49,50,52,53,54,55,58,60,62,63^ When studies were stratified by context of sample obtainment, the pooled prevalence was significantly higher among hospitalized individuals (48.2%; 95% CI, 36.4%-60.2%; τ^2^ = 0.62; *I*^2^ = 92.8%) than among nonhospitalized individuals (18.2%; 95% CI, 10.8%-29.1%; τ^2^ = 1.29; *I*^2^ = 95.4%) (*P* < .001) (Figure 3).

**Figure 4.**
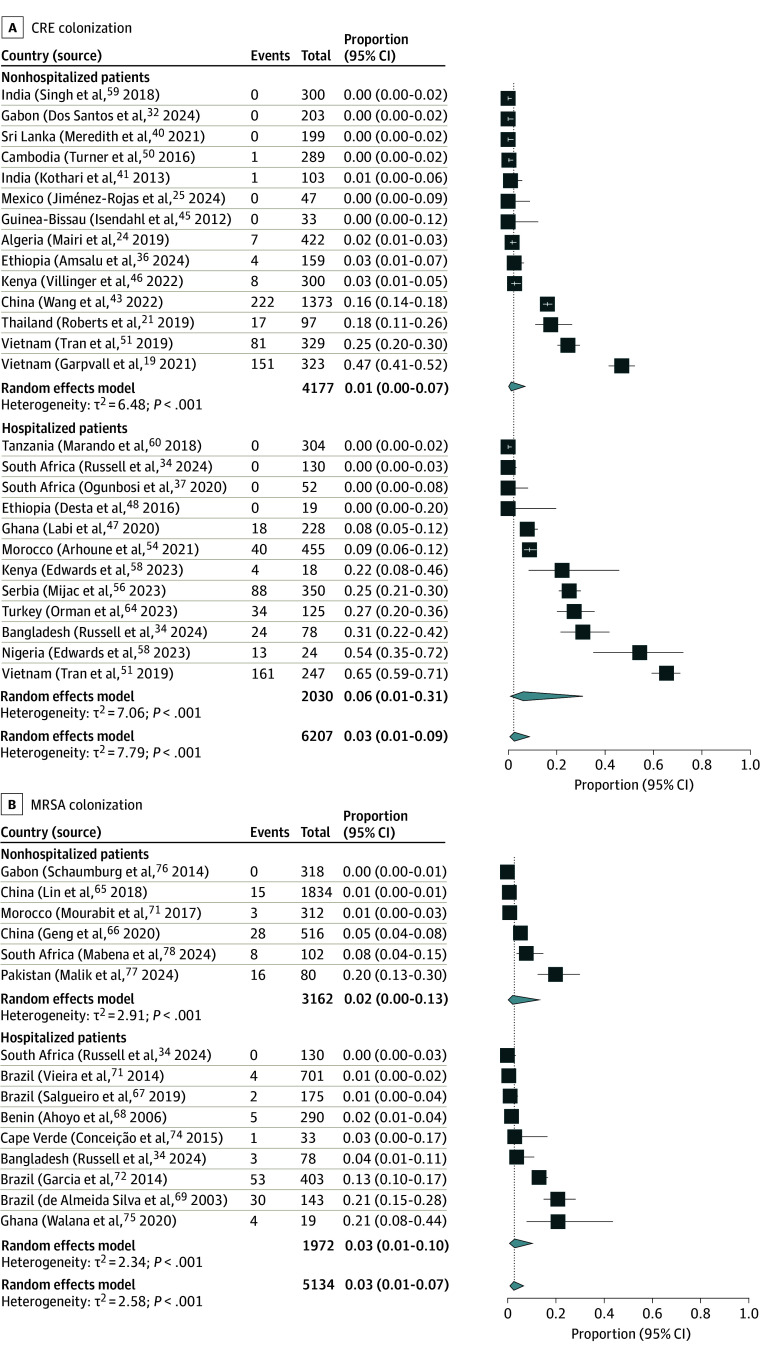
Meta-Analysis of Carbapenem-Resistant Enterobacterales (CRE) and Methicillin-Resistant *Staphylococcus aureus* (MRSA) Colonization Prevalence Stratified by Hospitalization Status

Based on 6207 individuals (53.6%) included in 26 studies (51.0%) (eTable 3 in Supplement 1), the pooled prevalence of CRE colonization among neonates and infants was 2.6% (95% CI, 0.7%-8.8%; τ^2^ = 7.79; *I^2^* = 95.6%).^19,21,24,25,32,34,36,37,40,41,43,45,46,47,48,50,51,54,56,58,59,60,64^ In the nonhospitalized subgroup, the pooled prevalence was 1.3% (95% CI, 0.2%-7.0%; τ^2^ = 6.48; *I*^2^ = 95.4%) compared with 6.3% (95% CI, 1.0%-30.8%; τ^2^ = 7.06; *I*^2^ = 95.4%) among hospitalized individuals (*P* = .16) (Figure 4A). Regional estimates were not calculated due to the limited number of studies in each region.

Among 31 studies (60.8%) reporting on the prevalence of 3GCRE colonization,^18,20,21,22,25,26,29,33,34,35,36,37,39,40,41,44,45,47,48,49,50,52,53,54,55,58,60,62,63^ 22 (71.0%) (eTable 4 in Supplement 1) provided data on the bacterial species isolated.^18,20,21,22,26,29,35,37,39,40,41,44,47,48,50,52,53,54,55,60,62,63^ Of 1723 isolates, *K pneumoniae* was the most frequently isolated microorganism, with a prevalence of 48.1% (95% CI, 35.1%-61.4%; τ^2^ = 1.30; *I*^2^ = 88.6%), followed by *E coli* (36.5%; 95% CI, 24.1%-50.1%; τ^2^ = 1.57; *I*^2^ = 92.0%) and *Enterobacter* species (1.0%; 95% CI, 0.2%-5.1%; τ^2^ = 6.10; *I*^2^ = 71.5%).

Ten studies (19.6%) reported data on isolated carbapenem-resistant bacterial species (eTable 4 in Supplement 1).^21,24,37,41,47,50,51,54,56,59^ Among 407 isolates, the most frequently isolated microorganism was again *K pneumoniae* (81.3%; 95% CI, 23.7%-98.4%; τ^2^ = 10.16; *I*^2^ = 66.2%) followed by *E coli* (5.6%; 95% CI, 0.5%-39.5%; τ^2^ = 5.93; *I*^2^ = 68.4%).

Twelve studies (23.5%) examined genes associated with 3GC resistance in 1052 isolates, with the most frequently identified gene group being CTXM-1.^35,37,39,41,45,53,54,58,60,61,62^ Eight studies (15.7%) investigated carbapenemase genes in 467 isolates, with the most frequently identified genes being *bla*_NDM_ and *bla*_OXA-48_ (eTable 4 in Supplement 1).^24,37,43,54,56,58,59^

To investigate the heterogeneity among the included studies, we computed multivariate meta-regression models to examine how study-level characteristics were associated with the prevalence of CRE colonization across the different studies (eTable 5 in Supplement 1). The prevalence of 3GCRE colonization was found to be significantly associated with the context (odds ratio [OR], 2.38 [95% CI, 1.09-5.21] for hospitalized individuals compared with nonhospitalized individuals) and time (OR, 3.12 [95% CI, 1.42-6.85] for after vs before 3 days of age) of sample obtainment. Overall, 52% (pseudo-*R*^2^) of the between-study variance could be explained by the model. The prevalence of CRE colonization was associated with study region (OR, 15.01 [95% CI, 2.17-103.84] when the study was conducted in the Asia-Pacific region compared with other regions) and the year of study conduct (OR, 8.50 [95% CI, 1.26-57.29] after 2017 compared with before 2017). Overall, these factors included in the model explained 57% (pseudo-*R*^2^) of the between-study variance.

**Figure 5.**
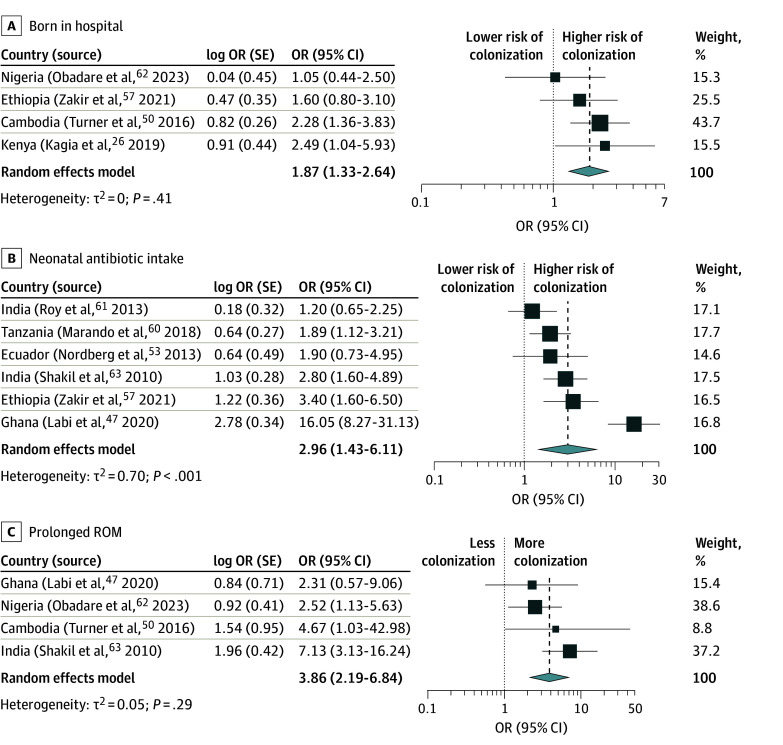
Meta-Analysis of Factors Associated With Third-Generation Cephalosporin–Resistant Enterobacterales Colonization OR indicates odds ratio; ROM, rupture of membranes.

Of the 19 studies that reported risk factor analysis,^17,21,24,26,28,38,40,43,47,50,53,54,56,57,59,60,61,62,63^ 11 (57.9%) reported factors associated with 3GCRE colonization,^17,21,26,47,50,53,57,60,61,62,63^ 5 (26.3%) with CRE colonization,^24,28,43,56,59^ and 3 (15.8%) with multidrug-resistant Enterobacterales colonization ^38,40,54^ (eTable 6 in Supplement 1). Seven factors that were associated with 3GCRE colonization and were reported in at least 3 studies were quantitatively synthesized (Figure 5 and eFigure 1 in Supplement 1). Hospital birth vs community health center or home birth (pooled OR, 1.87; 95% CI, 1.33-2.64; τ^2^ = 0.00; *I*^2^ = 0.0%), antibiotic use in the neonatal period (pooled OR, 2.96; 95% CI, 1.43-6.11; τ^2^ = 0.70; *I*^2^ = 86.4%), and prolonged rupture of membranes (ROM) (pooled OR, 3.86; 95% CI, 2.19-6.84; τ^2^ = 0.05; *I*^2^ = 20.7%) were significantly associated with colonization.

Thirty studies (58.8%) had low risk of bias^17,18,21,22,23,24,25,26,28,29,32,34,35,37,38,39,40,46,47,50,51,53,54,57,60,61,62,63^; 20 (39.2%), moderate risk of bias^19,20,27,30,31,33,36,41,42,44,45,48,49,52,55,56,58,59,64^; and 1 (2.0%), high risk of bias^43^ (eAppendix 2 in Supplement 1). The test for publication bias was not significant for either 3GCRE (*t* = 1.14; *P* = .26) or CRE (*t* = 0.69; *P* = .50). Sensitivity analyses yielded similar results to the main analysis (eFigure 2 in Supplement 1).

### MRSA Colonization

A total of 1352 studies pertaining to MRSA were identified, of which 16 studies (5580 individuals reported in 15 articles^34,65,66,67,68,69,70,71,72,73,74,75,76,77,78^) were included (Figure 1). The most represented WHO region was Africa (7 studies [43.8%]; 1204 individuals [21.6%]) (Figure 2).^34,68,71,74,75,76,78^ Almost all studies took place in an urban setting only (14 [87.5%]),^34,65,66,67,68,69,70,71,72,73,75,77,78^ and most studies recruited hospitalized patients (10 [62.5%])^34,67,68,69,70,72,73,74,75^ (eTable 7 in Supplement 1).

The main sample type was nasal swab only (8 studies [50.0%])^66,67,68,70,71,74,75,77^ followed by a nasal swab plus another sample site in 7 studies (43.8%)^34,65,69,72,76,78^ or a fecal swab (1 study [6.3%]).^73^ The latter was excluded from the meta-analysis but retained for descriptive purposes. Microbiologic techniques are detailed in eTable 3 and described in eAppendix 3 in Supplement 1.

Based on 5134 individuals included in 15 studies,^34,65,66,67,68,69,70,71,72,73,74,75,76,77,78^ the pooled prevalence of MRSA colonization was 2.7% (95% CI, 1.0%-6.7%; τ^2^ = 2.58; *I*^2^ = 93.5%). When the studies were stratified by context of sample obtainment, the prevalence of MRSA colonization was 2.2% (95% CI, 0.3%-13.2%; τ^2^ = 2.91; *I*^2^ = 94.6%) among nonhospitalized individuals and 3.1% (95% CI, 0.8%-10.3%; τ^2^ = 2.34; *I*^2^ = 91.2%) among hospitalized individuals (*P* = .72).

In the multivariate meta-regression model, the prevalence of MRSA colonization (eTable 5 in Supplement 1) was associated with studies conducted after 2012 (OR, 9.39; 95% CI, 1.04-84.58) and hospitalization in a neonatal intensive care unit (OR, 9.41; 95% CI, 1.05-84.48). This model accounted for 35% (pseudo-*R*^2^) of the between-study variance.

Only 2 studies reported risk factor analysis (eTable 8 in Supplement 1).^69,72^ Five studies (31.3%) were deemed to have a moderate risk of bias,^67,73,74,75,78^ while 11 (68.8%) were considered to have a low risk^34,65,66,68,69,70,71,72,76,77^ (eAppendix 2 in Supplement 1). The Peters test for publication bias was not significant (*t* = 1.13; *P* = .28).

## Discussion

This study found that a substantial proportion of infants younger than 3 months in LMICs were colonized with antibiotic-resistant critical pathogens. The overall pooled prevalence estimates were 30.2% for 3GCRE colonization, 2.6% for CRE colonization, and 2.7% for MRSA colonization, although with substantial heterogeneity. These pooled prevalence rates fall within the range of those reported among adults despite a limited exposure period. The pooled prevalence of maternal carriage of extended-spectrum β-lactamase (ESBL)–producing Enterobacterales in LMICs, as reported in a published meta-analysis, peaked at 50% in Asia and 30% in Africa.^79^ Additionally, a pooled estimate of 5.4% (95% CI, 3.7-7.4) has been reported for the prevalence of carbapenem-resistant *K pneumoniae* colonization in the general population.^80^ A meta-analysis of the prevalence of MRSA colonization in pregnant women^81^ found estimates ranging from 2.1% in low-income countries to 9.8% in countries with lower-middle income.

Considering the lack of substantial evidence for the hypothesis of in utero bacterial colonization, the neonatal microbiome at birth can be conceptualized as an empty ecological niche. Microbial diversity, initially characterized as low,^82^ gradually increases to reach adult levels by the age of 3 years.^83^ This limited diversity may facilitate the establishment of antibiotic-resistant pathogens in neonates even after brief exposure. Additionally, it may lead to faster bacterial selection following antibiotic administration,^84^ potentially increasing the effect of antibiotic administration on the acquisition of antibiotic-resistant pathogens in neonates compared with adults or children. In our analysis, early antibiotic administration was associated with a higher likelihood of 3GCRE colonization, highlighting the importance of preventing inappropriate antibiotic use in neonates and infants. Furthermore, the infant gut microbiota exhibits a higher proportion of Enterobacterales and a lower proportion of obligate anaerobes compared with the adult gut microbiota^85^ due to the oxygen-rich environment in the neonatal gut, potentially increasing susceptibility to gut colonization with 3GCRE or CRE. Similarly, *S aureus* nasal colonization is highest in the first few months of life and decreases thereafter, mainly due to competition with *Streptococcus pneumoniae*.^86^

Beyond the higher susceptibility to antibiotic-resistant pathogens that may be attributed to the neonatal microbiome, the precise contribution of the different transmission routes of these pathogens to neonates remains uncertain. Vertical transmission is often presumed to be the primary source for 3GCRE.^87^ Our study found a significant association between prolonged ROM and 3GCRE colonization, suggesting that prolonged ROM may be associated with increased likelihood of vertical transmission through longer exposure to maternal intestinal flora. However, it is important to recognize that transmission may also result from the hospital environment or health care workers, as neonates after extended ROM often require medical interventions such as resuscitation. Our findings revealed that sample obtainment after 3 days of life was associated with higher 3GCRE colonization rates, suggesting that a significant proportion of antibiotic-resistant bacteria are acquired through horizontal transmission. Additionally, being born in a hospital was associated with 3GCRE colonization. Thus, health care settings might be considered as a reservoir for antibiotic-resistant pathogens, especially in LMICs, where hygiene measures are often inadequate. In addition, hospitalization was associated with higher prevalence rates of 3GCRE and CRE colonization in the meta-regression models, supporting the pivotal role of health care–associated acquisition.

The prevalence of MRSA colonization was not associated with hospitalization status in subgroup analyses. This finding is consistent with a study indicating that neonatal acquisition of MRSA was common outside health care settings.^88^ However, in our meta-regression model, hospitalization in the neonatal intensive care unit (NICU) was a study-level factor associated with higher levels of MRSA carriage, which may reflect high circulation in NICUs. Surprisingly, our search yielded no studies investigating risk factors for neonatal MRSA acquisition in community settings. In high-income countries, a strong correlation has been suggested between maternal and neonatal carriage.^89^ Yet, the mode of transmission—whether vertically during childbirth or postnatally, possibly through breastfeeding—remains unclear and warrants further investigation.

Our study found that *K pneumoniae* was the most frequently identified species among neonates and infants colonized with a 3GCRE isolate. In contrast, ESBL *E coli* fecal carriage is often reported as the primary driver of 3GCRE spread in the adult community.^90^ *K pneumoniae* regardless of its resistance pattern is less prevalent than *E coli* at any age.^91^ However, it has been found that the proportion of neonates and infants colonized with *K pneumoniae* is higher compared with adults.^82^ In the neonatal microbiome, there is a competition between these 2 species,^91^ with various factors playing a role in these interactions. For instance, preterm birth and cesarean delivery may be associated with increased *K pneumoniae* colonization compared with *E coli* colonization.^91,92^ Additional research is required to establish whether our result may be attributed to factors favoring the colonization of 3GC-resistant *K pneumoniae* over 3GC-resistant *E coli* aside from increased exposure or gut colonization capacity. Furthermore, *K pneumoniae* is a major contributor to infant mortality in LMICs,^15^ being the most frequently isolated bacterial pathogen in several studies of neonatal sepsis.^93^ Given its virulence, specific prevention strategies targeting this bacteria species, such as vaccines (which are currently under development^94^), could be of valuable interest.

Our multivariate meta-regression revealed a significant association between the prevalence of CRE colonization and the year in which the study was conducted after adjustment for context of sample obtainment and region. This finding is consistent with the global spread of CRE in recent years.^95^ This trend mirrors the trajectory observed for 3GCRE,^90^ indicating that the prevalence of 3GCRE and/or CRE colonization at the time is likely higher than our results suggest.

Future studies should include larger numbers of participants and provide longitudinal follow-up, notably to better understand the dynamics of infant colonization. Given the increasing prevalence of AMR, these studies need to be conducted regularly to provide updated data. Molecular characterization of isolated strains, which was rarely performed in the included studies, could provide valuable insights into the circulation and transmission routes among neonates. Such information is pivotal for developing evidence-based interventions aimed at reducing the acquisition of antibiotic-resistant bacteria. Health care settings and neonatal antibiotic administration appear to be important factors in the acquisition of antibiotic-resistant bacteria, highlighting the importance of strengthening infection control and antimicrobial stewardship in maternity and neonatal units in LMICs.

### Limitations

Our study has several limitations. First, the small sample size in many studies may have led to a type of bias known as sampling fluctuations, potentially affecting the robustness of our findings. Due to the limited number of studies available and their geographic diversity, we were unable to provide accurate regional estimates. In addition, several studies were rejected during the review process because they relied on laboratory data without associated clinical data. Furthermore, some studies only reported colonization with multidrug-resistant Enterobacterales without specifying the 3GC resistance rate. These findings highlight missed opportunities and the need for standardization in the epidemiologic surveillance of AMR.

The high degree of heterogeneity is a major limitation of our study. This is supported by both the τ^2^ values and the *I*^2^ values, which suggest that most of the heterogeneity reflects true variations rather than random fluctuations. This is not surprising, as methodologic differences among studies as well as significant variations in AMR prevalence over time, across regions, and in different contexts contributed to this variability. Consequently, our findings should be interpreted with caution and considered as a general overview rather than definitive statistics. Meta-regression models were developed to identify the main determinants of heterogeneity for each pathogen of interest. It is important to note that generalizable conclusions should not be made without considering these parameters, such as the context of sample obtainment or study year. However, our meta-regression models were limited in addressing the between-study heterogeneity due to the lack of essential information needed to fully explain the variation in prevalence. For instance, data on infection prevention and control protocols, antibiotic use, or stewardship programs were rarely provided and often inconsistent across the included studies, making it challenging to include these crucial parameters in our analysis.

In addition, significant differences in microbiologic methods were noted among the included studies. For instance, selective preenrichment broth, recommended to enhance sensitivity,^96^ was inconsistently used prior to primary plating. Similarly, several studies screening for 3GCRE or CRE did not perform primary plating on culture media supplemented with 3GC or carbapenem, respectively, potentially reducing sensitivity, especially with low inoculum level. Various antibiotic disks were used to detect carbapenem resistance despite evidence indicating that ertapenem displays increased sensibility.^97^

## Conclusions

This systematic review and meta-analysis found that the carriage rate of antibiotic-resistant pathogens among individuals aged 0 to 3 months was high in LMICs. This study may provide a basis for further epidemiologic studies and the design of targeted interventions to prevent neonatal colonization with antibiotic-resistant bacteria in LMICs.

## Notes

Supplement 1. **eAppendix 1.** Full Search Terms for PubMed Database **eAppendix 2.** Supplementary Methods **eAppendix 3.** Description of Microbiologic Techniques **eFigure 1.** Forest Plots of Odds Ratios for 3GCRE Colonization Risk **eFigure 2.** Sensitivity Analyses **eTable 1.** JBI Tool and Results **eTable 2.** Description of Studies Included for 3GCRE Colonization **eTable 3.** Description of Studies Included for CRE Colonization **eTable 4.** Methods and Results of Studies Reporting Data on Bacterial Species or Resistance Genes **eTable 5.** Meta-Regression Models **eTable 6.** Description of Risk Factors Investigated and Main Findings of Studies Reporting Risk Factor Analysis for 3GCRE or CRE Colonization **eTable 7.** Description of Studies Included for MRSA Prevalence **eTable 8.** Description of Risk Factors Investigated and Main Findings of Studies Reporting Risk Factor Analysis for MRSA Colonization
eReferences

Supplement 2.
Data Sharing Statement
