# Supplementary material for: Neonatal Colonization With Antibiotic-Resistant Pathogens in Low- and Middle-Income Countries: A Systematic Review and Meta-Analysis
Source: JAMA Netw Open. 2024 Nov 5;7(11):e2441596. doi: 10.1001/jamanetworkopen.2024.41596 (PMC11581591; doi:10.1001/jamanetworkopen.2024.41596)
Supplement: Supplement 1. — eAppendix 1. Full Search Terms for PubMed Database eAppendix 2. Supplementary Methods eAppendix 3. Description of Microbiologic Techniques eFigure 1. Forest Plots of Odds Ratios for 3GCRE Colonization Risk eFigure 2. Sensitivity Analyses eTable 1. JBI Tool and Results eTable 2. Description of Studies Included for 3GCRE Colonization eTable 3. Description of Studies Included for CRE Colonization eTable 4. Methods and Results of Studies Reporting Data on Bacterial Species or Resistance Genes eTable 5. Meta-Regression Models eTable 6. Description of Risk Factors Investigated and Main Findings of Studies Reporting Risk Factor Analysis for 3GCRE or CRE Colonization eTable 7. Description of Studies Included for MRSA Prevalence eTable 8. Description of Risk Factors Investigated and Main Findings of Studies Reporting Risk Factor Analysis for MRSA Colonization eReferences [file jamanetwopen-e2441596-s001.pdf]

Supplementary Online Content

Beaumont AL, Kermorvant-Duchemin E, Breurec S, Huynh BT. Neonatal colonization with antibiotic-resistant pathogens in low- and middle-income countries: a systematic review and meta-analysis. *JAMA Netw Open*. 2024;7(10):e2441596.
doi:10.1001/jamanetworkopen.2024.41596

eAppendix 1. Full Search Terms for PubMed Database

eAppendix 2. Supplementary Methods

eAppendix 3. Description of Microbiologic Techniques

eFigure 1. Forest Plots of Odds Ratios for 3GCRE Colonization Risk

eFigure 2. Sensitivity Analyses

eTable 1. JBI Tool and Results

eTable 2. Description of Studies Included for 3GCRE Colonization

eTable 3. Description of Studies Included for CRE Colonization

eTable 4. Methods and Results of Studies Reporting Data on Bacterial Species or Resistance Genes

eTable 5. Meta-Regression Models

eTable 6. Description of Risk Factors Investigated and Main Findings of Studies Reporting Risk Factor Analysis for 3GCRE or CRE Colonization

eTable 7. Description of Studies Included for MRSA Prevalence

eTable 8. Description of Risk Factors Investigated and Main Findings of Studies Reporting Risk Factor Analysis for MRSA Colonization

eReferences

This supplementary material has been provided by the authors to give readers additional information about their work.

© 2024 Beaumont AL et al. *JAMA Network Open*.

eAppendix 1: Search strategy for PubMED database.

- ***Enterobacterales section***

("2000/01/01"[Date - Entry] : "2024/07/29"[Date - Entry])

AND

(Enterobacteriaceae [MH] OR Escherichia coli [MH] OR Klebsiella pneumoniae [MH] OR « Coliform Bacilli » [TW] OR « Enterobacteria* » [TW] OR « E. coli » [TW] OR « K. pneumoniae » [TW] OR « Gram-Negative » [TW] OR « Enterobacterales» [TW] OR "Escherichia coli" [TW] OR "Klebsiella pneumoniae")

AND

(beta-Lactam Resistance [MH] OR Carbapenem-Resistant Enterobacteriaceae [MH] OR Drug Resistance, Microbial [MH] OR « ESBL » [TW] OR « Extended-spectrum beta-lactamase* » [TW] OR « beta-lactamase » [TW] OR « Third generation cephalosporin resistant » [TW] OR « Multi-drug resistant » [TW] OR « Multidrug resistant » [TW] OR « Antimicrobial-resistant » [TW] OR « Antibiotic-resistant » [TW] OR « Carbapenem-Resistant » [TW])

AND

(Infant, Newborn[MH] OR « newborn » [TW] OR "neonate*"[TW] OR "neonatal")

AND

(Developing countries [MH] OR "Afghanistan"[TW] OR "Albania"[TW] OR "Algeria"[TW] OR "American Samoa"[TW] OR "Angola"[TW] OR "Armenia"[TW] OR "Azerbaijan"[TW] OR "Bangladesh"[TW] OR "Belarus"[TW] OR "Belize"[TW] OR "Benin"[TW] OR "Bhutan"[TW] OR "Bolivia"[TW] OR "Bosnia"[TW] OR "Herzegovina"[TW] OR "Hercegovina"[TW] OR "Bosnia-Herzegovina"[TW] OR "Botswana"[TW] OR "Brazil"[TW] OR "Bulgaria"[TW] OR "Burkina Faso"[TW] OR "Burundi"[TW] OR "Cabo Verde"[TW] OR "Cape Verde"[TW] OR "Cambodia"[TW] OR "Cameroon"[TW] OR "Central African Republic"[TW] OR "Chad"[TW] OR "China"[TW] OR "Colombia"[TW] OR "Comoros"[TW] OR "Costa Rica"[TW] OR "Cote d'Ivoire"[TW] OR "Ivory Coast"[TW] OR "Cuba"[TW] OR "Democratic republic of the Congo"[TW] OR "Congo"[TW] OR "Zaire"[TW] OR "DRC"[TW] OR "Democratic People's Republic Korea"[TW] OR "North Korea"[TW] OR "Djibouti"[TW] OR "Dominica"[TW] OR "Dominican Republic"[TW] OR "Ecuador"[TW] OR "Egypt"[TW] OR "El Salvador"[TW] OR "Salvador"[TW] OR "Eritrea"[TW] OR "Eswatini"[TW] OR "Swaziland"[TW] OR "Ethiopia"[TW] OR "Fiji"[TW] OR "Gabon"[TW] OR "Gambia"[TW] OR "Georgia"[TW] OR "Ghana"[TW] OR "Grenada"[TW] OR "Guatemala"[TW] OR "Guinea"[TW] OR "Guinea-Bissau"[TW] OR "Guyana"[TW] OR "Haiti"[TW] OR "Honduras"[TW] OR "India"[TW] OR "Indonesia"[TW] OR "Iran"[TW] OR "Iraq"[TW] OR "Jamaica"[TW] OR "Jordan"[TW] OR "Kazakhstan"[TW] OR "Kenya"[TW] OR "Kiribati"[TW] OR "Kosovo"[TW] OR "Kyrgyzstan"[TW] OR "Kirghizia"[TW] OR "Kyrgyz Republic"[TW] OR "Kirgizstan"[TW] OR "Kirghizstan"[TW] OR "Kyrghizstan"[TW] OR "Lao PDR"[TW] OR "Laos"[TW] OR "Lao People*"[TW] OR "Lebanon"[TW] OR "Lesotho"[TW] OR "Liberia"[TW] OR "Libya"[TW] OR "Madagascar"[TW] OR "Malawi"[TW] OR "Malaysia"[TW] OR "Maldives"[TW] OR "Mali"[TW] OR "Marshall Islands"[TW] OR "Mauritania"[TW] OR "Mauritius"[TW] OR "Mexico"[TW] OR "Micronesia"[TW] OR "Moldova"[TW] OR "Mongolia"[TW] OR "Montenegro"[TW] OR "Morocco"[TW] OR "Mozambique"[TW] OR "Myanmar"[TW] OR "Namibia"[TW] OR "Nepal"[TW] OR "Nicaragua"[TW] OR "Niger"[TW] OR "Nigeria"[TW] OR "North Macedonia"[TW] OR "Macedonia"[TW] OR "Pakistan"[TW] OR "Panama"[TW] OR "Papua New Guinea"[TW] OR "PNG"[Title/Abstract] OR "Paraguay"[TW] OR "Peru"[TW] OR "Philippines"[TW] OR "Republic of the Congo"[TW] OR "Russian Federation"[TW] OR "Russia"[TW] OR "Rwanda"[TW] OR "Samoa"[TW] OR "Sao Tome"[TW] OR "Principe"[TW] OR "Senegal"[TW] OR "Serbia"[TW] OR "Sierra Leone"[TW] OR "Solomon Islands"[TW] OR "Somalia"[TW] OR "South Africa"[TW] OR "South Sudan"[TW] OR "Sri Lanka"[TW] OR "St Lucia"[TW] OR "Saint Lucia"[TW] OR "St Vincent"[TW] OR "the Grenadines"[TW] OR "Saint Vincent"[TW] OR "Sudan"[TW] OR "Suriname"[TW] OR "Syria"[TW] OR "Syrian Arab Republic"[TW] OR "Tajikistan"[TW] OR "Tadjikistan"[TW] OR

© 2024 Beaumont AL et al. *JAMA Network Open*.

"Tadzhikistan"[TW] OR "Tanzania"[TW] OR "Thailand"[TW] OR "Timor-Leste"[TW] OR "Timor Leste"[TW] OR "Togo"[TW] OR "Tonga"[TW] OR "Tunisia"[TW] OR "Turkey"[TW] OR "Turkmenistan"[TW] OR "Tuvalu"[TW] OR "Uganda"[TW] OR "Ukraine"[TW] OR "Uzbekistan"[TW] OR "Vanuatu"[TW] OR "Vietnam"[TW] OR "West Bank"[TW] OR "Gaza"[TW] OR "Palestine"[TW] OR "Yemen"[TW] OR "Zambia"[TW] OR "Zimbabwe"[TW] OR "Antigua"[TW] OR "Barbuda"[TW] OR "Argentina"[TW] OR "Barbados"[TW] OR "Chili"[TW] OR "Croatia"[TW] OR "Czech Republic"[TW] OR "Czechia"[TW] OR "Equatorial Guinea"[TW] OR "Estonia"[TW] OR "Gibraltar"[TW] OR "Hungary"[TW] OR "Latvia"[TW] OR "Lithuania"[TW] OR "Malta"[TW] OR "Mauritius"[TW] OR "Nauru"[TW] OR "Northern Mariana Islands"[TW] OR "Oman"[TW] OR "Panama"[TW] OR "Paulu"[TW] OR "Poland"[TW] OR "Porto Rico"[TW] OR "Romania"[TW] OR "Saudi Arabia"[TW] OR "Slovakia"[TW] OR "Trinidad"[TW] OR "Tobago"[TW] OR "Saint Kitts"[TW] OR "St Kitts"[TW] OR "Nevis"[TW] OR "Uruguay"[TW] OR "Venezuela"[TW])

NOT

(sheep[TW] OR calves[TW] or bovine[TW])

- ➔ **MRSA section**

("2000/01/01"[Date - Entry] : "2023/06/01"[Date - Entry])

AND

(« Staphylococcus aureus » [TW] OR « Staphylococcus aureus » [MH] OR « S.aureus » [TW] OR “microbiota” [TW] OR « screening » [TW] OR « carrier » [TW] OR « carriage » [TW] OR « colonisation » [TW] OR « colonization » [TW] OR « colonised » [TW] OR « colonized »)

AND

(Drug Resistance, Microbial [MH] OR Drug Resistance, Microbial [TW] OR« Multi-drug resistant » [TW]] OR « carbapenem resistant » [TW] OR « Multidrug resistant » [TW] OR « Antimicrobial-resistant » [TW] OR « Antibiotic-resistant » [TW] OR « antibiotic susceptibility »[TW] OR « antibiotic susceptibility »[TW] OR Methicillin-Resistant Staphylococcus aureus[MH] OR Methicillin-Resistant Staphylococcus aureus [TW] OR « MRSA » [TW])

AND

(Infant, Newborn[MH] OR « newborn » [TW] OR "neonate*"[TW] OR "neonatal"[TW])

AND

(Developing countries [MH] OR Developing countries [TW] OR "Afghanistan"[TW] OR "Albania"[TW] OR "Algeria"[TW] OR "American Samoa"[TW] OR "Angola"[TW] OR "Armenia"[TW] OR "Azerbaijan"[TW] OR "Bangladesh"[TW] OR "Belarus"[TW] OR "Belize"[TW] OR "Benin"[TW] OR "Bhutan"[TW] OR "Bolivia"[TW] OR "Bosnia"[TW] OR "Herzegovina"[TW] OR "Hercegovina"[TW] OR "Bosnia-Herzegovina"[TW] OR "Botswana"[TW] OR "Brazil"[TW] OR "Bulgaria"[TW] OR "Burkina Faso"[TW] OR "Burundi"[TW] OR "Cabo Verde"[TW] OR "Cape Verde"[TW] OR "Cambodia"[TW] OR "Cameroon"[TW] OR "Central African Republic"[TW] OR "Chad"[TW] OR "China"[TW] OR "Colombia"[TW] OR "Comoros"[TW] OR "Costa Rica"[TW] OR "Cote d’Ivoire"[TW] OR "Ivory Coast"[TW] OR "Cuba"[TW] OR "Democratic republic of the Congo"[TW] OR "Congo"[TW] OR "Zaire"[TW] OR "DRC"[TW] OR "Democratic People’s Republic Korea"[TW] OR "North Korea"[TW] OR "Djibouti"[TW] OR "Dominica"[TW] OR "Dominican Republic"[TW] OR "Ecuador"[TW] OR "Egypt"[TW] OR "El Salvador"[TW] OR "Salvador"[TW] OR "Eritrea"[TW] OR "Eswatini"[TW] OR "Swaziland"[TW] OR "Ethiopia"[TW] OR "Fiji"[TW] OR "Gabon"[TW] OR "Gambia"[TW] OR "Georgia"[TW] OR "Ghana"[TW] OR "Grenada"[TW] OR "Guatemala"[TW] OR "Guinea"[TW] OR "Guinea-Bissau"[TW] OR "Guyana"[TW] OR "Haiti"[TW] OR "Honduras"[TW] OR "India"[TW] OR "Indonesia"[TW] OR "Iran"[TW] OR "Iraq"[TW] OR "Jamaica"[TW] OR "Jordan"[TW] OR "Kazakhstan"[TW] OR "Kenya"[TW] OR "Kiribati"[TW] OR "Kosovo"[TW] OR "Kyrgyzstan"[TW] OR "Kirghizia"[TW] OR "Kyrgyz Republic"[TW] OR "Kirgizstan"[TW] OR "Kirghizstan"[TW] OR "Kyrghizstan"[TW] OR "Lao PDR"[TW] OR "Laos"[TW] OR "Lao People*"[TW] OR "Lebanon"[TW] OR "Lesotho"[TW] OR "Liberia"[TW] OR "Libya"[TW] OR "Madagascar"[TW] OR

© 2024 Beaumont AL et al. *JAMA Network Open*.

"Malawi"[TW] OR "Malaysia"[TW] OR "Maldives"[TW] OR "Mali"[TW] OR "Marshall Islands"[TW] OR "Mauritania"[TW] OR "Mauritius"[TW] OR "Mexico"[TW] OR "Micronesia"[TW] OR "Moldova"[TW] OR "Mongolia"[TW] OR "Montenegro"[TW] OR "Morocco"[TW] OR "Mozambique"[TW] OR "Myanmar"[TW] OR "Namibia"[TW] OR "Nepal"[TW] OR "Nicaragua"[TW] OR "Niger"[TW] OR "Nigeria"[TW] OR "North Macedonia"[TW] OR "Macedonia"[TW] OR "Pakistan"[TW] OR "Panama"[TW] OR "Papua New Guinea"[TW] OR "PNG"[Title/Abstract] OR "Paraguay"[TW] OR "Peru"[TW] OR "Philippines"[TW] OR "Republic of the Congo"[TW] OR "Russian Federation"[TW] OR "Russia"[TW] OR "Rwanda"[TW] OR "Samoa"[TW] OR "Sao Tome"[TW] OR "Principe"[TW] OR "Senegal"[TW] OR "Serbia"[TW] OR "Sierra Leone"[TW] OR "Solomon Islands"[TW] OR "Somalia"[TW] OR "South Africa"[TW] OR "South Sudan"[TW] OR "Sri Lanka"[TW] OR "St Lucia"[TW] OR "Saint Lucia"[TW] OR "St Vincent"[TW] OR "the Grenadines"[TW] OR "Saint Vincent"[TW] OR "Sudan"[TW] OR "Suriname"[TW] OR "Syria"[TW] OR "Syrian Arab Republic"[TW] OR "Tajikistan"[TW] OR "Tadjikistan"[TW] OR "Tadzhikistan"[TW] OR "Tanzania"[TW] OR "Thailand"[TW] OR "Timor-Leste"[TW] OR "Timor Leste"[TW] OR "Togo"[TW] OR "Tonga"[TW] OR "Tunisia"[TW] OR "Turkey"[TW] OR "Turkmenistan"[TW] OR "Tuvalu"[TW] OR "Uganda"[TW] OR "Ukraine"[TW] OR "Uzbekistan"[TW] OR "Vanuatu"[TW] OR "Vietnam"[TW] OR "West Bank"[TW] OR "Gaza"[TW] OR "Palestine"[TW] OR "Yemen"[TW] OR "Zambia"[TW] OR "Zimbabwe"[TW] OR "Antigua"[TW] OR "Barbuda"[TW] OR "Argentina"[TW] OR "Barbados"[TW] OR "Chili"[TW] OR "Croatia"[TW] OR "Czech Republic"[TW] OR "Czechia"[TW] OR "Equatorial Guinea"[TW] OR "Estonia"[TW] OR "Gibraltar"[TW] OR "Hungary"[TW] OR "Latvia"[TW] OR "Lithuania"[TW] OR "Malta"[TW] OR "Mauritius"[TW] OR "Nauru"[TW] OR "Northern Mariana Islands"[TW] OR "Oman"[TW] OR "Panama"[TW] OR "Paulu"[TW] OR "Poland"[TW] OR "Porto Rico"[TW] OR "Romania"[TW] OR "Saudi Arabia"[TW] OR "Slovakia"[TW] OR "Trinidad"[TW] OR "Tobago"[TW] OR "Saint Kitts"[TW] OR "St Kitts"[TW] OR "Nevis"[TW] OR "Uruguay"[TW] OR "Venezuela"[TW])

NOT

(sheep[TW] OR calves[TW] or bovine[TW])

eAppendix 2: Supplementary methods

Risk of bias calculation

For each of the 8 criteria of the tool, 'yes' scored 1, 'no' and 'unclear' scored 0. Where the authors did not provide a specific calculation for sample size adequacy, we estimated it using prevalence data on colonisation in the general population in each WHO region[1–3]. An overall score (out of 8) was calculated for each study. We considered studies with a score above 7 as having a low risk of bias, 5 or 6 as having a moderate risk of bias, and below 5 as having a high risk of bias.

Multiple meta-regression

Multiple meta-regression analyses were performed to account for potential sources of heterogeneity, using the R package metafor. Variables were included in the model hierarchically, guided by a scientific rationale to minimise multicollinearity. Model fitness was assessed using the pseudo-R-squared statistic[4].

Risk factors meta-analysis

Risk factors for colonisation with each type of drug-bug pair, specifically 3GC-R-E, carbapenem-R-E, multidrug-resistant[5] Enterobacterales or MRSA were extracted to create a descriptive table. Variables documented in at least 3 studies were quantitatively synthesised by pooling pre-calculated crude odds ratios (after logarithmic transformation), using random-effects models with inverse variance method. Results are reported as unadjusted odds ratio (OR) estimates and CIs (95% CI), and Paule-Mandel estimator for τ-squared statistic. *p*-values below 0.05 are considered statistically significant.

Analysis of bacterial species

The proportions of *E. coli*, *K. pneumoniae* and *Enterobacter spp* were calculated for each study, using either the total number of 3GC-R-E isolates or CRE isolates as the denominator. A random-effects model was used to pool these percentages, using the same parameters as above.

© 2024 Beaumont AL et al. *JAMA Network Open*.

eAppendix 3: Description of microbiological techniques.

For the detection of 3GC-R-E (eTable 1), the main microbiological screening technique (23/39, 59%) was culture on selective media containing 3GC followed by antibiotic susceptibility testing (AST) through disc diffusion. Thirteen studies cultured samples on non-selective media and detected resistance by disc diffusion. In contrast, two studies did not perform a culture step and directly used PCR testing. One study did not specify the technique employed. A pre-enrichment broth was used in four studies. Twenty-four studies specified the method used to identify extended-spectrum beta-lactamase (ESBL) phenotypic activity, 15 used PCR to identify resistance genes and four studies performed whole-genome sequencing (WGS).

For the detection of CRE (eTable 2), two studies used a molecular-based approach (PCR directly on the sample without a culture step) and 28 a culture-based approach. Among these, five performed an enrichment broth before primary plating. Eleven used a culture media enriched with carbapenem and 15 a non-selective media or enriched with 3GC (not mentioned in 2). AST was performed through disk diffusion in 16 studies and microbroth dilution (Vitek2 mainly) in nine. Sixteen studies used at least one complementary phenotypic testing method, nine used PCR to screen for carbapenemase genes and five studies performed WGS on a sample of isolates.

For the detection of MRSA, four studies used a pre-enrichment broth, none of which was supplemented with antibiotics. The sample was cultured on a hypersaline agar medium in nine cases, on blood agar in five cases, on a selective medium with antibiotic in two cases and one study a chromogenic medium. MRSA was mostly identified using resistance to oxacillin or cefoxitin by disc diffusion (14/16), with additional PCR in seven cases. One study used PCR only (not specified in one case).

© 2024 Beaumont AL et al. *JAMA Network Open*.

eFigure 1: Residual forest plots of odds ratio for risk of 3GC-R-E colonisation (complementary to Figure 5).

A. Forest plot of odds ratio of colonization with 3GC-R-E associated with pre-term birth.

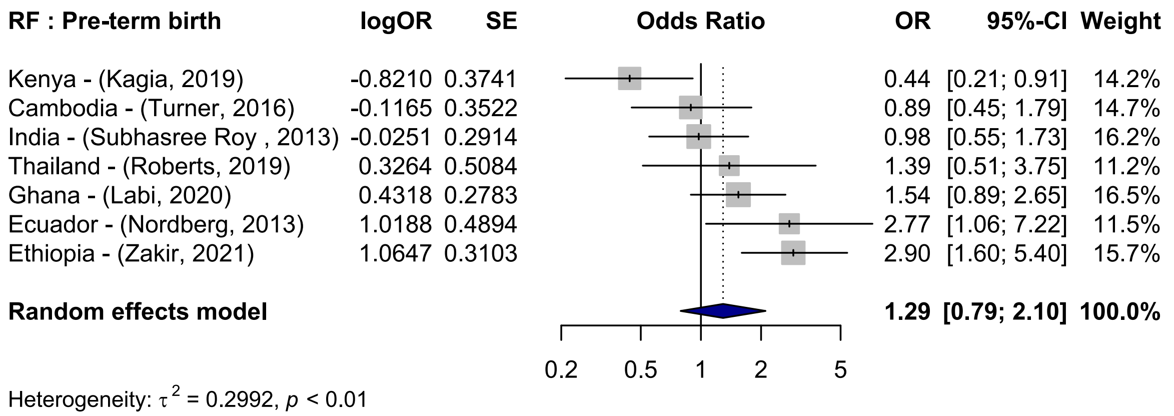

B. Forest plot of odds ratio of colonization with 3GC-R-E associated with low-birth weight.

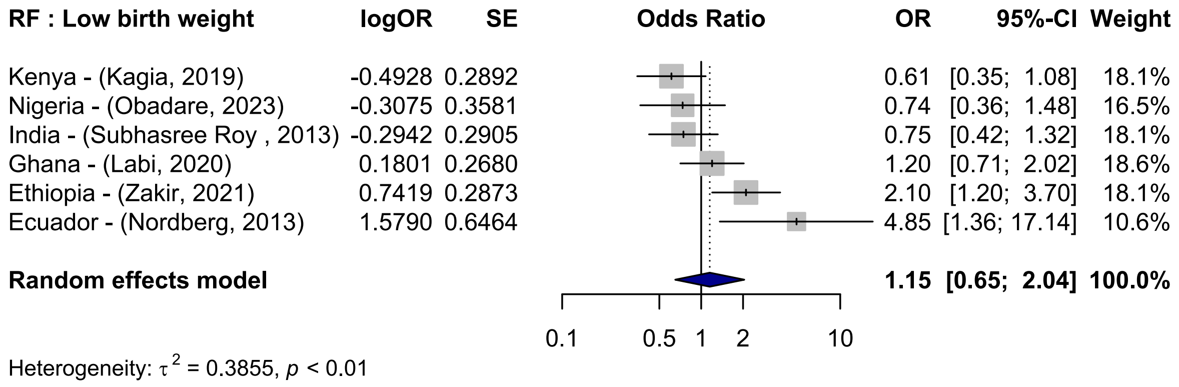

C. Forest plot of odds ratio of colonization with 3GC-R-E associated with breastfeeding.

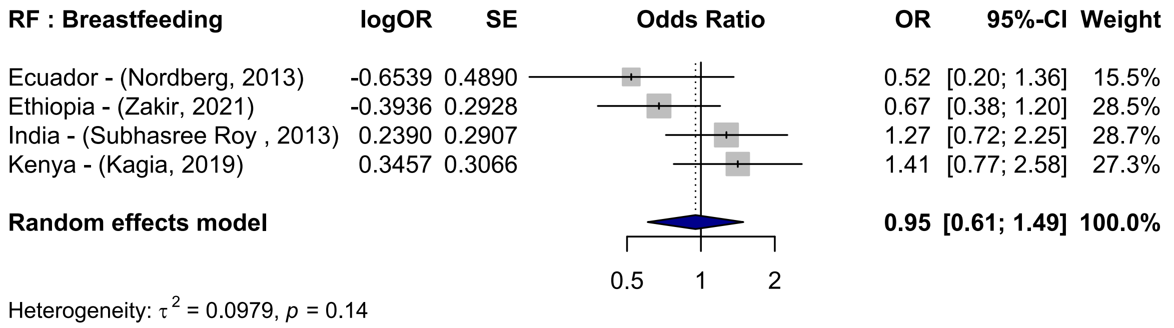

D. Forest plot of odds ratio of colonization with 3GC-R-E associated with C-section.

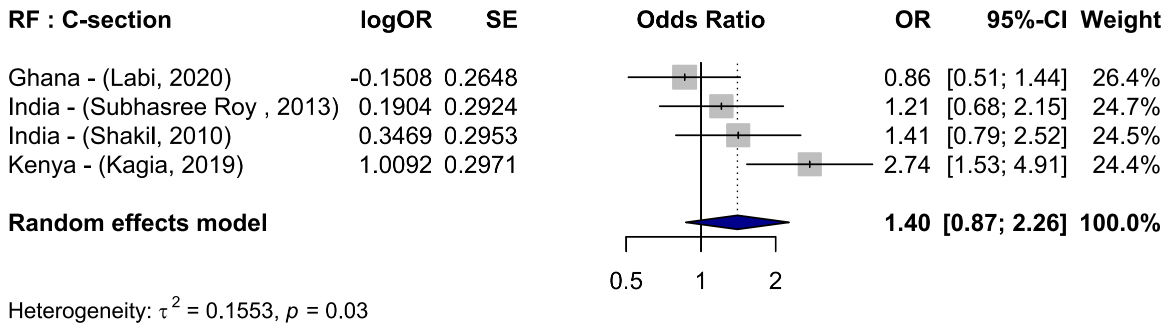

© 2024 Beaumont AL et al. *JAMA Network Open*.

eFigure 2: Sensitivity analyses.

A. Forest plot of included studies for 3GC-resistant *Enterobacterales* colonization prevalence, stratified by sampling context, after exclusion of two studies without culture step.

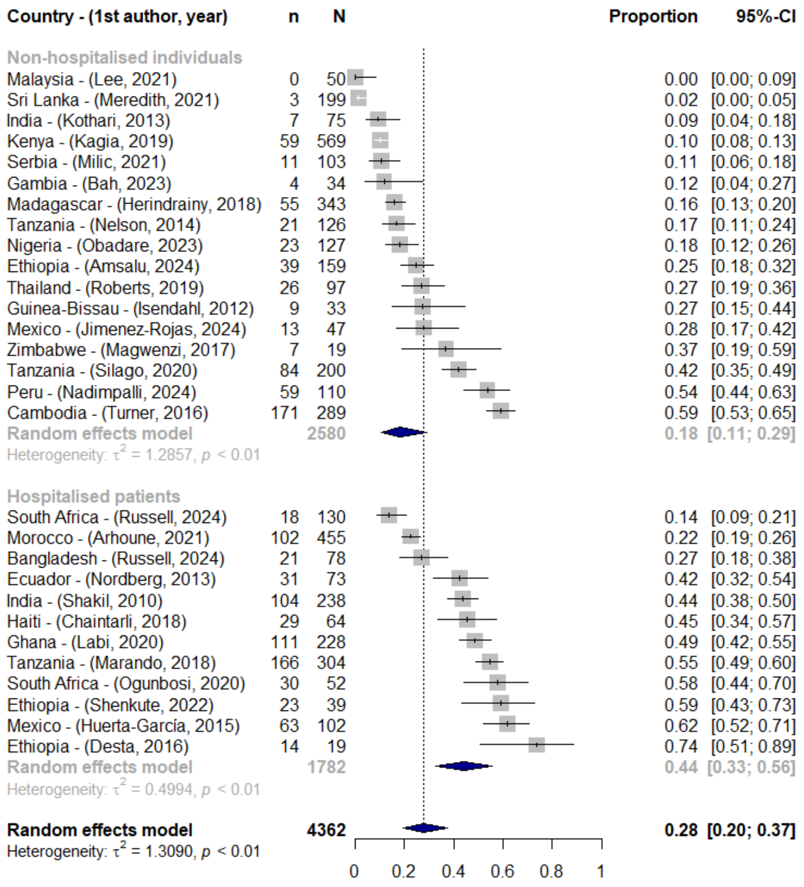

© 2024 Beaumont AL et al. *JAMA Network Open*.

B. **Forest plot of included studies for carbapenem-resistant *Enterobacterales* colonization prevalence, stratified by sampling context, after exclusion of two studies without culture step.**

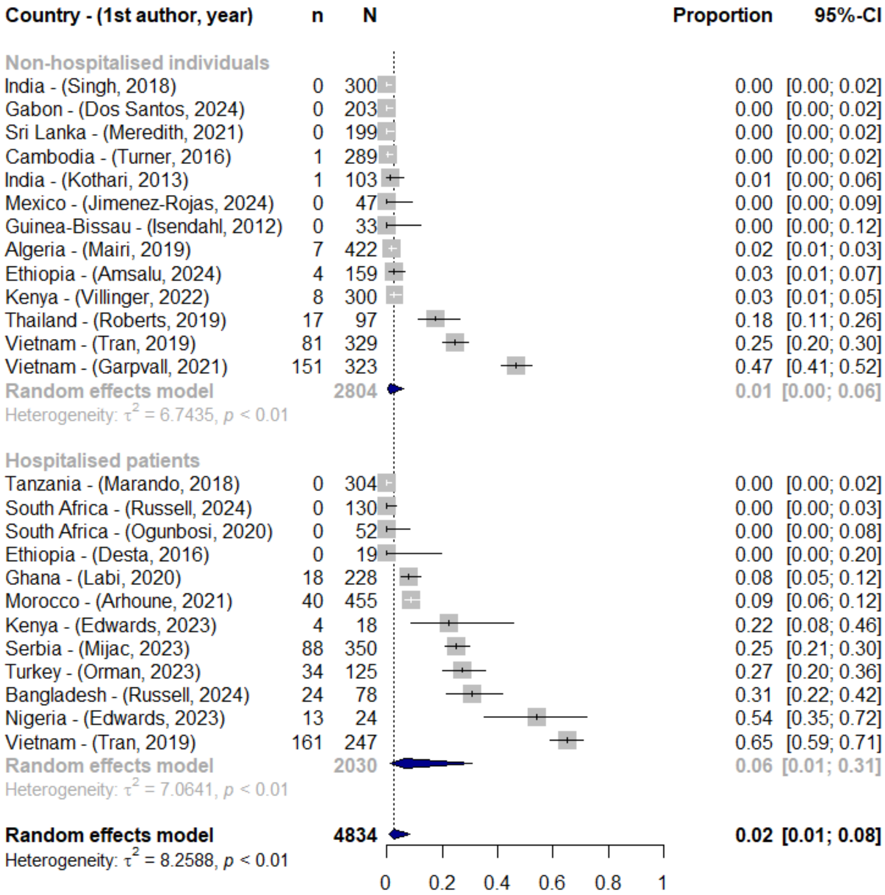

© 2024 Beaumont AL et al. *JAMA Network Open*.

C. Forest plot of included studies for 3GC-resistant *Enterobacterales* colonization prevalence, stratified by sampling context, after exclusion of one study with a high risk of bias.

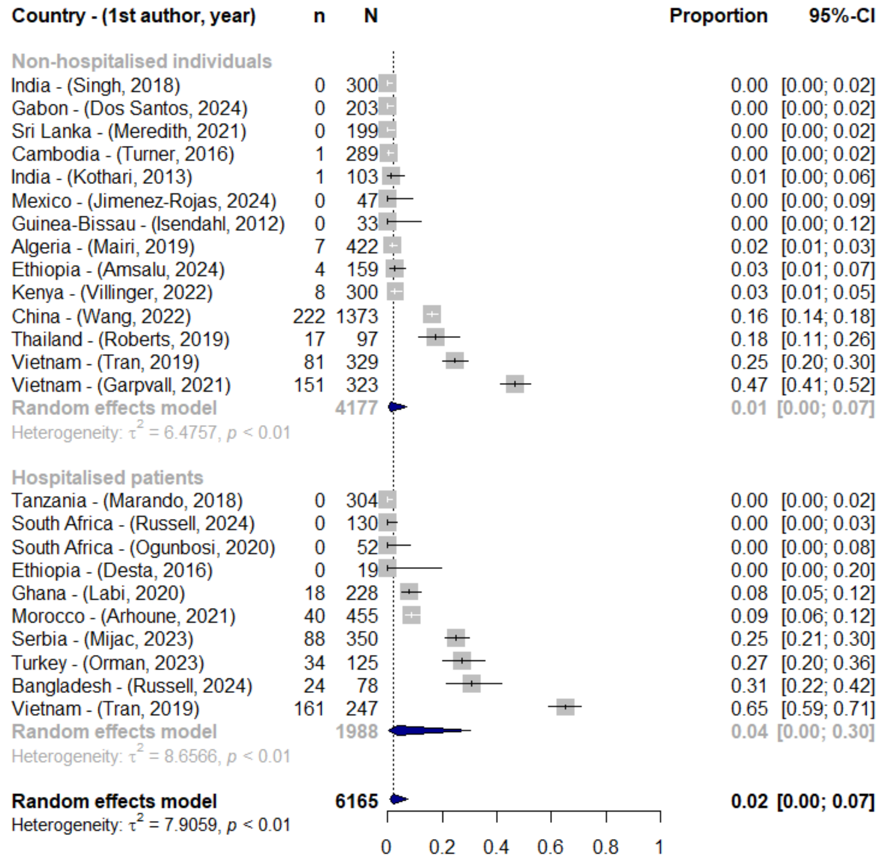

© 2024 Beaumont AL et al. *JAMA Network Open*.

eTable 1: Joanna Briggs Institute (JBI) tool and results.

A. **JBI tool and interpretation.**

Question stated in official JBI tool	Interpretation for this review
1. Was the sample frame appropriate to address the target population?	<=> appropriate and explicit inclusion/exclusion criteria (clearly stated and replicable)
2. Were study participants recruited in an appropriate way?	<=> clearly stated and appropriate recruitment method (consecutive patient, or random sample)
3. Was the sample size adequate?	<=> if sample size was not calculated by the authors, please refer to estimation made thanks from data from the literature
4. Were the study subjects and the setting described in detail?	<=> data on health status, age, gender distribution (gestational age, low weight, antibiotics)
5. Was data analysis conducted with sufficient coverage of the identified sample?	We chose not to include this question as it pertains to the exclusion of certain subgroups within the general population. Our review is already focused on a specific subgroup.
6. Were valid methods used for the identification of the condition?	<=> appropriate sample (at least rectal swab / stool sample for C3G-R or carbapenem-R gram-negative bacteria screening ; at least one skin swab for MRSA screening)
7. Was the condition measured in a standard, reliable way for all participants?	<=> valid microbiological methods (detailed description of the method and adequation to national or international guidelines)
8. Was there appropriate statistical analysis?	<=> numerator and denominator clearly reported
9. Was the response rate adequate, and if not, was the low response rate managed appropriately?	<=> reasonable number of refusals/dropouts and clear report

B. **Results of risk bias assessment for studies reporting data on 3GC-R and/or carbapenem-R colonization among neonates**

study_id	Question n°1	Question n°2	Question n°3	Question n°4	Question n°6	Question n°7	Question n°8	Question n°9	Risk of bias
Zimbabwe - (Magwenzi, 2017)	1	1	0	0	1	1	1	0	Moderate

© 2024 Beaumont AL et al. *JAMA Network Open*.

study_id	Question n°1	Question n°2	Question n°3	Question n°4	Question n°6	Question n°7	Question n°8	Question n°9	Risk of bias
Ecuador - (Nordberg, 2013)	1	1	0	1	1	1	1	1	Low
Cambodia - (Turner, 2016)	1	1	1	1	1	1	1	1	Low
India - (Subhasree Roy , 2013)	1	1	1	0	1	1	1	1	Low
Mexico - (Huerta-García, 2015)	1	1	1	0	1	1	1	0	Moderate
Vietnam - (Garpvall, 2021)	1	1	1	0	1	0	0	1	Moderate
Madagascar - (Herindrainy, 2018)	1	1	1	1	1	1	1	1	Low
Thailand - (Roberts, 2019)	1	1	0	1	1	1	1	1	Low
India - (Singh, 2018)	1	1	1	0	1	0	1	1	Moderate
Algeria - (Mairi, 2019)	1	1	1	0	1	1	1	1	Low
Kenya - (Kagia, 2019)	1	1	1	1	1	1	1	1	Low
Sri Lanka - (Meredith, 2021)	1	1	1	1	1	1	1	1	Low
Serbia - (Mijac, 2023)	0	1	1	0	1	1	1	1	Moderate
Kenya - (Villinger, 2022)	1	1	1	1	1	1	1	1	Low
India - (Kothari, 2013)	1	1	0	0	1	0	1	1	Moderate

© 2024 Beaumont AL et al. *JAMA Network Open*.

study_id	Question n°1	Question n°2	Question n°3	Question n°4	Question n°6	Question n°7	Question n°8	Question n°9	Risk of bias
Ghana - (Labi, 2020)	1	1	1	1	1	1	1	1	Low
Serbia - (Milic, 2021)	1	1	1	1	1	1	1	1	Low
Tanzania - (Nelson, 2014)	1	1	0	0	1	1	1	1	Moderate
Morocco - (Arhoune, 2021)	1	1	1	1	1	1	1	1	Low
Haiti - (Chaintarli, 2018)	1	1	0	0	1	1	1	1	Moderate
Ethiopia - (Desta, 2016)	1	1	0	0	1	1	1	1	Moderate
Vietnam - (Tran, 2019)	1	1	1	1	1	1	1	1	Low
Ethiopia - (Zakir, 2021)	1	1	1	1	1	1	1	1	Low
Ethiopia - (Shenkute, 2022)	1	1	0	0	1	1	1	1	Moderate
Malaysia - (Lee, 2021)	1	1	0	1	1	1	1	1	Low
Turkey - (Orman, 2023)	0	1	1	1	0	0	1	1	Moderate
China - (Wang, 2022)	0	0	1	0	1	1	0	1	High
Tanzania - (Silago, 2020)	1	1	1	1	1	0	1	1	Low
Vietnam - (Tran, 2019)	1	1	1	0	1	1	1	1	Low
Brazil - (Sakai, 2020)	1	1	1	1	1	1	1	1	Low
Guinea-Bissau - (Isendahl, 2012)	1	1	0	0	1	1	1	1	Moderate

© 2024 Beaumont AL et al. *JAMA Network Open*.

study_id	Question n°1	Question n°2	Question n°3	Question n°4	Question n°6	Question n°7	Question n°8	Question n°9	Risk of bias
South Africa - (Ogunbosi, 2020)	1	1	0	1	1	1	1	1	Low
Thailand - (Hetzer, 2019)	1	1	0	1	0	1	1	1	Moderate
Turkey - (Akturk, 2016)	1	1	1	0	1	1	1	1	Low
India - (Shakil, 2010)	1	1	1	1	1	1	1	1	Low
Kenya - (Edwards, 2023)	1	0	0	1	1	1	0	1	Moderate
Nigeria - (Edwards, 2023)	1	0	0	1	1	1	0	1	Moderate
Tanzania - (Marando, 2018)	1	1	1	1	1	1	1	1	Low
Nigeria - (Obadare, 2023)	1	1	1	1	1	1	1	1	Low
Malaysia - (Boo, 2005)	1	1	1	1	1	1	1	1	Low
Ethiopia - (Amsalu, 2024)	0	1	1	1	1	1	0	1	Moderate
Gambia - (Bah, 2023)	1	1	0	1	0	0	1	1	Moderate
Gabon - (Dos Santos, 2024)	0	1	1	1	1	1	1	1	Low
Mexico - (Jimenez-Rojas, 2024)	1	1	1	1	1	1	1	1	Low
China - (Ma, 2014)	1	1	0	1	1	1	1	1	Low

© 2024 Beaumont AL et al. *JAMA Network Open*.

study_id	Question n°1	Question n°2	Question n°3	Question n°4	Question n°6	Question n°7	Question n°8	Question n°9	Risk of bias
Kenya - (Mogeni, 2024)	1	1	0	0	1	1	1	1	Moderate
Peru - (Nadimpalli, 2024)	1	1	1	1	1	1	1	1	Low
Cameroon - (Njeuna, 2024)	1	1	0	1	0	1	1	1	Moderate
South Africa - (Russell, 2024)	1	1	1	1	1	1	1	1	Low
Bangladesh - (Russell, 2024)	1	1	1	1	1	1	1	1	Low
Kenya - (Rwigi, 2024)	1	1	0	0	1	1	1	1	Moderate

C. **Results of risk bias assessment for studies reporting data on MRSA colonization among neonates**

study_id	Question n°1	Question n°2	Question n°3	Question n°4	Question n°6	Question n°7	Question n°8	Question n°9	Risk of bias
Ghana - (Walana, 2020)	1	1	0	0	1	1	1	1	Moderate
Brazil - (Silva, 2003)	1	1	1	0	1	1	1	1	Low
Brazil - (Vieira, 2014)	1	1	1	1	1	1	1	1	Low
Gabon - (Schaumburg, 2013)	1	1	1	1	1	1	1	1	Low
China - (Geng, 2020)	1	1	1	1	1	1	1	1	Low

© 2024 Beaumont AL et al. *JAMA Network Open*.

study_id	Question n°1	Question n°2	Question n°3	Question n°4	Question n°6	Question n°7	Question n°8	Question n°9	Risk of bias
China - (Lin, 2018)	1	1	1	1	1	1	1	1	Low
China - (Ai, 2020)	1	0	1	0	1	1	1	1	Moderate
Benin - (Ahoyo, 2006)	1	1	1	0	1	1	1	1	Low
Brazil - (Garcia, 2014)	1	0	1	1	1	1	1	1	Low
Morocco - (Mourabit , 2017)	1	0	1	1	1	1	1	1	Low
Cape Verde - (Aires-De-Sousa, 2015)	1	1	0	0	1	1	1	1	Moderate
Brazil - (Salgueiro, 2019)	1	1	1	0	1	1	1	0	Moderate
Pakistan - (Malik, 2023)	1	1	1	1	1	1	0	1	Low
South Africa - (Mabena, 2024)	1	1	0	1	1	0	1	1	Moderate
South Africa - (Russell, 2024)	1	1	1	1	1	1	1	1	Low
Bangladesh - (Russell, 2024)	1	1	1	1	1	1	1	1	Low

© 2024 Beaumont AL et al. *JAMA Network Open*.

eTable 2: Description of studies included for 3GC-R-E colonisation.

Study ID	Inclu-ded in m-a[1]	WHO re-gion	WB cat.[2]	Special pop.	Design	Con text[3]	Hospital and ward structure	IPC measures[4]	ATB[5]	Bacteria	Pre-enrichment broth	3GC resistance detection technique	N	n	Individual estimated prevalence
Bangladesh - (Russell, 2024)	yes	SEAR	LM	yes_low birth weight	cross-sectional study nested in a pilot RCT	H	Children's hospital	according to the trial protocol (NeoCHG)		Entero-bacteria	None	culture on MacConkey agar + AST (disc diffusion, CLSI)	78	21	26.9% [18.3-37.7]
Brazil - (Sakai, 2020)	No (prevalence not extractible)	AMR	UM	no	cross-sectional study	H	Neonatal unit from a University Hospital, 30 beds (10 beds NICU, 20 beds intermediate care unit)	Mothers can remain full-time and are encouraged to perform skin-to-skin contact	368/433 (85%)	Entero-bacteria	None	Bacteriological medium used unclear + disc diffusion (CLSI)	443		
Cambodia - (Turner, 2016)	yes	WPR	L	no	cohort study	NH				Entero-bacteria	None	MacConkey agar plate with cefpodoxime disk + diffusion method (CLSI) + double-disc synergy test	289	171	59.2% [53.4-64.7]
Cameroon - (Njeuna, 2024)	No (naso-pharynx swab)	AFR	LM	no	cross-sectional study	NH				Ecoli+Kp	None	CHROMagar ESBL + AST (EUCAST, CLSI) + PCR for ESBL genes	90	5	
Ecuador - (Nordberg, 2013)	yes	AMR	UM	no	cohort study	H	NICU with 400 admissions/year, four rooms, with 25 beds in total	Average patient-to-nurse ratio: 7:1. 1 sink, chlorhexidine/alcohol hand disinfectant and gloves available in each room. Gloves not used routinely when in	51/73 (69%)	Entero-bacteria	None	MacConkey agar plate with 1 mg/L cefotaxime & 1mg/L ceftazidime + Vitek 2 AST (EUCAST) + double-disc synergy test +	73	31	42.5% [31.8-53.9]

© 2024 Beaumont AL et al. *JAMA Network Open*.

Study ID	Inclu-ded in m-a[1]	WHO re-gion	WB cat.[2]	Special pop.	Design	Con text [3]	Hospital and ward structure	IPC measures[4]	ATB[5]	Bacteria	Pre-enrichment broth	3GC resistance detection technique	N	n	Individual estimated prevalence
								contact with neonates. Textile gowns used by staff members and changed every day. Parents not allowed inside.				PCR for ESBL genes			
Ethiopia - (Amsalu, 2024)	yes	AFR	L	no	cohort study nested in a larger cohort	NH			9/159 (5,6%)	Entero-bacteria	None	Culture on colorex™ESBL + AST (MicroScan, CLSI) + PCR for ESBL genes	159	39	24.5% [18.5-31.8]
Ethiopia - (Desta, 2016)	yes	AFR	L	no	cross-sectional study	H	Neonatal unit of University Teaching hospital of 500 beds (largest hospital in Ethiopia)			Entero-bacteria	None	CHROMagar ESBL + Vitek2 + Neo-Sensitabs/ROSCO kits	19	14	73.7% [50.9-88.5]
Ethiopia - (Shenkute, 2022)	yes	AFR	L	no	cross-sectional study	H	NICU of a 200-bed hospital (covering 2 million of people)			Entero-bacteria	None	MacConkey agar plate + disk diffusion (CLSI) + double-disc synergy test	39	23	59% [43.4-72.9]
Ethiopia - (Zakir, 2021)	No (first sample data not extractible)	AFR	L	no	cross-sectional study	H	NICU of a General Hospital, with 32 beds (two rooms of 16). Usually 600 admissions/year		206/212 (97,2%) during hospitalisation	Entero-bacteria	None	MacConkey agar plate + disk diffusion (CLSI) + double-disc synergy test	212	72	
Gambia - (Bah, 2023)	yes	AFR	L	<2000g	cohort study	NH				Entero-bacteria	None	Culture (CLSI) + identification of genes on WGS (unclear)	34	4	11.8% [4.1-27.2]
Ghana - (Labi, 2020)	yes	AFR	LM	no	cross-sectional study	H	NICU of Teaching hospital (KBTH): 55 beds (3 cubicles for high-dependency care	Routine screening for MDRGN carriage not standard	118/228 (51,8%)	Entero-bacteria	None	MacConkey agar plate + disk-	228	111	48.7% [42.3-55.1]

© 2024 Beaumont AL et al. *JAMA Network Open*.

Study ID	Inclu-ded in m-a[1]	WHO re-gion	WB cat.[2]	Special pop.	Design	Con text[3]	Hospital and ward structure	IPC measures[4]	ATB[5]	Bacteria	Pre-enrichment broth	3GC resistance detection technique	N	n	Individual estimated prevalence
							and a 5-bed kangaroo mother care ward) and NICU of a military hospital (37MH): 20 beds (3 cubicles)	practice at the 2 hospitals.				diffusion + ROSCO kits			
Guinea-Bissau - (Isendahl, 2012)	yes	AFR	L	yes (tachycardia +/- fever)	cross-sectional study	NH				Entero-bacteria	None	ChromID ESBL + Vitek2 AST (EUCAST) + PCR for ESBL genes	33	9	27.3% [14.9-44.4]
Haiti - (Chaintarli, 2018)	yes	AMR	L	no	cross-sectional study	H	Neonatal unit of MSF obstetric emergency hospital		43/64 (69%)	Entero-bacteria	None	MacConkey plate + Vitek2 AST (CLSI) + double-disc synergy test	64	29	45.3% [33.7-57.4]
India - (Kothari, 2013)	yes	SEAR	LM	low-birth weight	cohort study	NH			0/75 (0%) - exclusion criteria	Entero-bacteria	yes, selective (trypticase soy broth with a meropenem disk)	MacConkey agar plate + disk-diffusion + PCR for ESBL genes	75	7	9.3% [4.3-18.3]
India - (Shakil, 2010)	yes	SEAR	LM	no	cohort study	H	NICU			Ecoli+Kp	None	Bacteriological medium used unclear + disc diffusion (CLSI) + double disc synergy test + MIC ceftazidim and/or cefotaxime test strips	238	104	43.7% [37.5-50.1]
India - (Subhasree Roy, 2013)	no (specific bacterial species)	SEAR	LM	no	cohort study	NH			137/210 (65%)	Ecoli	None	MacConkey agar plate + disk diffusion method (CLSI) +double-disc synergy test + MIC	210	103	

© 2024 Beaumont AL et al. *JAMA Network Open*.

Study ID	Inclu-ded in m-a[1]	WHO re-gion	WB cat.[2]	Special pop.	Design	Con text[3]	Hospital and ward structure	IPC measures[4]	ATB[5]	Bacteria	Pre-enrichment broth	3GC resistance detection technique	N	n	Individual estimated prevalence
												ceftazidim, cefotaxime and cefepime test strips			
Kenya - (Edwards, 2023)	yes	AFR	LM	no	cohort study	H	Neonatal unit of a Tertiary University Hospital, ~1200 admissions per year			Entero-bacteria	None	PCR for ESBL genes directly on rectal and/or stool samples	18	13	72.2% [48.8-87.8]
Kenya - (Kagia, 2019)	yes	AFR	LM	no	cohort study	NH				Entero-bacteria	None	MacConkey agar plate with gentamicin 8% and cefotaxime and ceftazidime disks + disk diffusion (CLSI) with double-disc method	569	59	10.4% [8.1-13.2]
Kenya - (Mogeni, 2024)	no (specific bacterial species)	AFR	LM	no	cross-sectional study nested in a RCT	H	2 Tertiary University Hospital (serving around 1,1 million people each)			Ecoli	yes (tryptic soy broth)	culture on MacConkey agar & Mueller-Hilton+ AST (CLSI) + double-disc synergy test + PCR for ESBL genes	12	1	
Kenya - (Rwigi, 2024)	no (specific bacterial species)	AFR	LM	no	cross-sectional study nested in a RCT	H	2 Tertiary University Hospital (serving around 1,1 million people each)			Klebsiella pneumoniae	yes (tryptic soy broth)	culture on MacConkey agar & Mueller-Hilton + AST (CLSI) + double-disc synergy test + PCR for ESBL genes	19	9	
Madagascar - (Herindrainy, 2018)	yes	AFR	L	no	cohort study	NH			5/343 (1,6%)	Entero-bacteria	None	CHROMagar ESBL + Double-disc synergy test (CA-SFM)	343	55	16% [12.5-20.3]

© 2024 Beaumont AL et al. *JAMA Network Open*.

Study ID	Inclu-ded in m-a[1]	WHO re-gion	WB cat.[2]	Special pop.	Design	Con text [3]	Hospital and ward structure	IPC measures[4]	ATB[5]	Bacteria	Pre-enrichment broth	3GC resistance detection technique	N	n	Individual estimated prevalence
Malaysia - (Boo, 2005)	No (case-control)	WPR	UM	no	case-control study	H	NICU of a University Hospital	Weekly rectal swab screening of all neonates for ESBL Klebsiella sp. Implemented since 1999		Klebsiella pneumoniae	None	MacConkey agar plate with 4 mg/L ceftazidime + double disc synergy test	369	81	
Malaysia - (Lee, 2021)	yes	WPR	UM	preterm	cohort	NH				Entero-bacteria	None	MacConkey agar with cefotaxime + disk diffusion (CLSI) with combined disc test + PCR for ESBL genes	50	0	1% [0-8.5]
Mexico - (Huerta-García, 2015)	yes	AMR	UM	no	cohort study	H	Neonatal units from 2 general hospitals (41 beds in hospital A, 96 beds in hospital B).	Hospital A: SICU of 35 incubators (15 cm between each), in a room with 2 sinks. NICU: 50 m2 area, 6 radiant warmers 50 cm apart. An alcohol-based hand sanitizer for hand hygiene. Hospital B: general care unit with 30 incubators and two SICUs with 30 incubators each (20 cm part). 1 hand-washing sink for every 10 incubators. NICU: 2 rooms with 3 radiant warmers, 50 cm apart.		Entero-bacteria	None	MacConkey agar with 1 mg/L ceftazidime + disc diffusion + synergy tests	102	63	61.8% [52.1-70.6]

© 2024 Beaumont AL et al. *JAMA Network Open*.

Study ID	Inclu-ded in m-a[1]	WHO re-gion	WB cat.[2]	Special pop.	Design	Con text[3]	Hospital and ward structure	IPC measures[4]	ATB[5]	Bacteria	Pre-enrichment broth	3GC resistance detection technique	N	n	Individual estimated prevalence
Mexico - (Jimenez-Rojas, 2024)	yes	AMR	UM	no	cohort study	NH			28/47 (59%)	Ecoli+Kp	None	culture on MacConkey agar + Vitek2 (CLSI) + Double-disc synergy test	47	13	27.7% [16.8-41.9]
Morocco - (Arhoune, 2021)	yes	EMR	LM	no	cohort study	H	NICU of a University Hospital, 18 beds divided into 2 sectors of 9 beds each (NICU and preterm unit)	3 seniors, 8 physicians, and 6 nurses are assigned daily	190/207(92%) during hospitalisation	Entero-bacteria	yes, non-selective (Brain Heart infusion)	MacConkey agar plate + disk-diffusion (EUCAST) + double-disc synergy test + PCR for ESBL genes	455	102	22.4% [18.8-26.5]
Nigeria - (Edwards, 2023)	yes	AFR	LM	no	cohort study	H	Neonatal unit of a Tertiary University Hospital, ~1000 admissions per year		1	Entero-bacteria	None	PCR for ESBL genes directly on rectal and/or stool samples	24	19	79.2% [59.1-91.2]
Nigeria - (Obadare, 2023)	yes	AFR	L	no	cohort study	NH			113/127 (89%)	Entero-bacteria	yes, selective (tryptic soy broth + vancomycin and/or cefpodoxime)	MacConkey agar plate with cefpodoxime + disk diffusion method (CLSI) + double-disc synergy test + PCR for ESBL genes	127	23	18.1% [12.3-25.8]
Peru - (Nadimpalli, 2024)	yes	AMR	UM	no	cohort study nested in a larger cohort	NH				Ecoli+Kp	yes, non-selective (tryptic soy broth)	CHROMagar ESBL + Double-disc synergy test +WGS (subset)	110	59	53.6% [44.4-62.7]
Serbia - (Milic, 2021)	yes	EUR	UM	preterm	cohort study	NH			100% (but after sampling)	Entero-bacteria	None	ChromID ESBL + disc diffusion (EUCAST) + double-disc synergy test +	103	11	10.7% [5.9-18.3]

© 2024 Beaumont AL et al. *JAMA Network Open*.

Study ID	Inclu-ded in m-a[1]	WHO re-gion	WB cat.[2]	Special pop.	Design	Con text[3]	Hospital and ward structure	IPC measures[4]	ATB[5]	Bacteria	Pre-enrichment broth	3GC resistance detection technique	N	n	Individual estimated prevalence
												PCR for ESBL genes			
South Africa - (Ogunbosi, 2020)	yes	AFR	UM	no	cross-sectional study	H	Pediatric unit of a 273-bedded tertiary university hospital			Entero-bacteria	None	ChromID ESBL + Vitek2 (CLSI) + PCR for ESBL genes	52	30	57.7% [44.2-70.1]
South Africa - (Russell, 2024)	yes	AFR	UM	yes_low birth weight	cross-sectional study nested in a pilot RCT	H	Tertiary Hospital	according to the trial protocol (NeoCHG)		Entero-bacteria	None	culture on MacConkey agar + AST (disc diffusion, CLSI)	130	18	13.8% [8.9-20.9]
Sri Lanka - (Meredith, 2021)	yes	SEAR	LM	no	cohort study	NH			3/199 (1,5%)	Entero-bacteria	None	MacConkey agar with 2 mg/L cefotaxime (CLSI) + decrease of ≥ 3 doubling dilutions in the MIC for either cefotaxime or ceftazidime tested in combination with 4 µg/mL clavulanic acid + PCR for ESBL genes + WGS	199	3	1.5% [0.3-4.5]
Tanzania - (Marando, 2018)	yes	AFR	L	signs of sepsis	cohort study	H	Neonatal unit of a Tertiary University Hospital		82/304 (27%)	Entero-bacteria	None	MacConkey agar with 2 mg/L cefotaxime (CLSI) + disc diffusion with synergy + Double-disc synergy test +WGS	304	166	54.6% [49-60.1]

© 2024 Beaumont AL et al. *JAMA Network Open*.

Study ID	Inclu-ded in m-a[1]	WHO re-gion	WB cat.[2]	Special pop.	Design	Con text [3]	Hospital and ward structure	IPC measures[4]	ATB[5]	Bacteria	Pre-enrichment broth	3GC resistance detection technique	N	n	Individual estimated prevalence
Tanzania - (Nelson, 2014)	yes	AFR	L	no	cohort study	NH			113/126 (89%)	Entero-bacteria	None	MacConkey agar plate with 2 mg/L cefotaxime + disk diffusion method (CLSI) + double-disc synergy test	126	21	16.7% [11.1-24.2]
Tanzania - (Silago, 2020)	yes	AFR	L	signs of sepsis	cross-sectional study	NH			175/200 (87,5%)	Entero-bacteria	None	MacConkey agar plate + disk diffusion method (CLSI) +double-disc synergy test	200	84	42% [35.4-48.9]
Thailand - (Hetzer, 2019)	no (specific bacterial species)	SEAR	LM	no	cohort study	NH			6,3% before 48h	Ecoli	None	MacConkey agar with cephalosporin + disc diffusion (EUCAST)	142	12	
Thailand - (Roberts, 2019)	yes	SEAR	LM	no	cohort study	NH				Entero-bacteria	None	CHROMagar ESBL + disk diffusion (CLSI) + double-disc synergy test	97	26	26.8% [19-36.4]
Zimbabwe - (Magwenzi, 2017)	yes	AFR	L	no	cohort study	NH				Entero-bacteria	yes, selective (nutrient broth with a cefpodoxime disc added to the bijou bottle)	MacConkey agar plate with cefpodoxime disk + ChromID ESBL + disk diffusion method (CLSI) + combined disc test	19	7	36.8% [19-59.1]

[1]m-a : included in meta-analysis of prevalence
[2]World Bank category, L : low-income country, LM : lower-middle income country, UM : upper-middle income country
[3]H : Hospitalised, N-H : Non-hospitalised
[4]IPC : infection prevention and control
[5]% of the study population receiving antibiotics, if extractible from the article
Empty grey cells indicate missing data (could not be extracted from the paper).or non-applicable information (i.e. hospital structure for community-based studies)

© 2024 Beaumont AL et al. *JAMA Network Open*.

eTable 3: Description of Studies Included for CRE Colonization.

Study ID	Included in m-a[1]	WHO region	WB cat.[2]	Spe-cial pop.	Design	Con text [3]	Hospital and ward structure	IPC measures [4]	ATB[5]	Bacteria	Pre-enrichme nt broth	Method for carbapenem-R detection	N	n	Individual estimated preva-lence
Algeria - (Mairi, 2019)	yes	AFR	LM	no	cohort study	NH				Entero-bacteria	yes, selective (trypticase soy broth supplemented with ertapenem)	MacConkey agar with 0.5 mg/L ertapenem + disc diffusion (CLSI) + PCR for carbapenemase genes	422	7	1.7% [0.7-3.5]
Bangladesh - (Russell, 2024)	yes	SEAR	LM	yes (low birth weight)	cross-sectional study nested in a pilot RCT	H	Children's hospital	according to the trial protocol (NeoCHG)		Entero-bacteria	None	culture on MacConkey agar + AST (disc diffusion, CLSI)	78	24	30.8% [21.6-41.8]
Brazil - (Sakai, 2020)	no (prevalence not extractible)	AMR	UM	no	cross-sectional study	H	Neonatal unit from a University Hospital, 30 beds (10 beds NICU, 20 beds intermediate care unit)	Mothers can remain full-time and are encouraged to perform skin-to-skin contact	368/ 433 (85%)	Entero-bacteria	None	Bacteriological medium used unclear + disk diffusion (CLSI)	433	1	
Cambodia - (Turner, 2016)	yes	WPR	L	no	cohort study	NH				Entero-bacteria	None	MacConkey agar with imipenem + disc diffusion (imipenem disc, CLSI)	289	1	0.3% [0-2.1]
China - (Ma, 2014)	No (case-control)	WPR	UM	no	case-control study	H	NICU of Union Hospital Beijing			Klebsiella pneu-moniae	None	culture on MH + AST (Vitek2, CLSI) + Modified Hodge Test	27		
China - (Wang, 2022)	yes	WPR	UM	no	cross-sectional study	NH				Entero-bacteria	None	EMB agar with meropenem + AST (Vitek2)	1,373	222	16.2% [14.3-18.2]
Ethiopia - (Amsalu, 2024)	yes	AFR	L	no	cohort study nested in a larger cohort	NH			9/159 (5,6%)	Entero-bacteria	None	Culture on MacConkey agar with imipenem & Colorex™mSuperCARBA™ + AST (Microscan, CLSI) + WGS	159	4	2.5% [0.8-6.5]

© 2024 Beaumont AL et al. *JAMA Network Open*.

Study ID	Included in m-a[1]	WHO region	WB cat.[2]	Spe-cial pop.	Design	Con text [3]	Hospital and ward structure	IPC measures [4]	ATB[5]	Bacteria	Pre-enrichment broth	Method for carbapenem-R detection	N	n	Individual estimated preva-lence
Ethiopia - (Desta, 2016)	yes	AFR	L	no	cross-sectional study	H	Neonatal unit of University Teaching hospital of 500 beds (largest hospital in Ethiopia)			Entero-bacteria	None	CHROMagar ESBL + Vitek 2 (EUCAST) + Neo-Sensitabs/ROSCO kits	19	0	2.5% [0-19.8]
Ethiopia - (Zakir, 2021)	no (first sample data not extractible)	AFR	L	no	cross-sectional study	H	NICU of a General Hospital, with 32 beds (two rooms of 16). Usually 600 admissions/year		206/ 212 (97%) during hospitalisation	Entero-bacteria	None	MacConkey agar plate + disk diffusion (ertapenem disk) + Modified Carbapenem Inactivation Method (mCIM)	212	5	
Gabon - (Dos Santos, 2024)	yes	AFR	UM	no	cohort study	NH			150/ 203 (74%)	Entero-bacteria	None	CHROMagar mSuperCARBA + MIC ertapenem + PCR + WGS	203	0	0.2% [0-2.2]
Ghana - (Labi, 2020)	yes	AFR	LM	no	cross-sectional study	H	NICU of Teaching hospital (KBTH): 55 beds (3 cubicles for high-dependency care and a 5-bed kangaroo mother care ward) and NICU of a military hospital (37MH): 20 beds (3 cubicles)	Routine screening for MDRGN carriage not standard practice at the 2 hospitals.	118/ 228 (52%)	Entero-bacteria	None	MacConkey agar plate + disk diffusion (meropenem disk) (EUCAST) + ROSCO kits + PCR for carbapenemase genes + WGS	228	18	7.9% [5-12.2]
Guinea-Bissau - (Isendahl, 2012)	yes	AFR	L	yes (tachycardia +/- fever)	cross-sectional study	NH				Entero-bacteria	None	ChromID ESBL-selective medium + disc diffusion (meropenem disk) (EUCAST) + MIC testing	33	0	1.5% [0-12.4]
India - (Kothari, 2013)	yes	SEAR	LM	low-birth weight	cohort study	NH			0/75 (0%) - exclusion criteria	Entero-bacteria	yes, selective (trypticase soy broth with a	MacConkey agar plate + disk diffusion (meropenem disk) + MIC testing + MBL production testing by EDTA E-test +	103	1	1% [0-5.8]

© 2024 Beaumont AL et al. *JAMA Network Open*.

Study ID	Included in m-a[1]	WHO region	WB cat.[2]	Spe-cial pop.	Design	Con text [3]	Hospital and ward structure	IPC measures [4]	ATB[5]	Bacteria	Pre-enrichme nt broth	Method for carbapenem-R detection	N	n	Individual estimated preva-lence
											meropenem disk)	Modified Hodge Test + PCR for carbapenemase genes			
India - (Singh, 2018)	yes	SEAR	LM	no	cohort study	NH			64/300 (21%)	Entero-bacteria	yes, selective (trypticase soy broth containing ertapenem disk)	MacConkey agar plate + disc diffusion (ertapenem/meropenem disks) (CLSI) + MHT + PCR for carbapenemase genes	300	0	0.2% [0-1.5]
Kenya - (Edwards, 2023)	yes	AFR	LM	no	cohort study	H	Neonatal unit of a Tertiary University Hospital, ~1200 admissions per year			Entero-bacteria	None	PCR for carbapenemase genes directly on rectal and/or stool sample	18	4	22.2% [8.5-45.7]
Kenya - (Villinger, 2022)	yes	AFR	LM	no	cohort study	NH				Entero-bacteria	None	Selective chromogenic ESBL agar + Vitek2 AST (CLSI) +Imipenem E-test for confirmation + WGS	300	8	2.7% [1.3-5.3]
Mexico - (Jimenez-Rojas, 2024)	yes	AMR	UM	no	cohort study	NH			28/47 (59%)	Ecoli+Kp	None	culture on MacConkey agar + Vitek2 (CLSI)	47	0	1% [0-9]
Morocco - (Arhoune, 2021)	yes	EMR	LM	no	cohort study	H	NICU of a University Hospital, 18 beds divided into 2 sectors of 9 beds each (NICU and preterm unit)	3 seniors, 8 physicians, and 6 nurses are assigned daily	190/207 (92%) during hospitali sation	Entero-bacteria	yes, non-selective (Brain Heart infusion)	MacConkey agar plate + disc diffusion (ertapenem disk) + MHT + PCR for carbapenemase genes	455	40	8.8% [6.5-11.8]
Nigeria - (Edwards, 2023)	yes	AFR	LM	no	cohort study	H	Neonatal unit of a Tertiary University Hospital, ~1000 admissions per year		100%	Entero-bacteria	None	PCR for carbapenemase genes directly on rectal and/or stool sample	24	13	54.2% [35.1-72.1]
Serbia - (Mijac, 2023)	yes	EUR	UM	Pre-term	cohort study	H	Neonatal unit in a tertiary hospital, 700-900 admissions per year. Placement of UVC is performed if	Alcohol-based combinations are used on skin, medical devices and surfaces, quaternary ammonium +	100% (Protoco l for all patients on admissi on: blood	Entero-bacteria	None	ChromID Carba/chromID OXA-48 + disk diffusion (EUCAST) + PCR for carbapenemase genes	350	88	25.1% [20.9-29.9]

© 2024 Beaumont AL et al. *JAMA Network Open*.

Study ID	Included in m-a[1]	WHO region	WB cat.[2]	Spe-cial pop.	Design	Con text [3]	Hospital and ward structure	IPC measures [4]	ATB[5]	Bacteria	Pre-enrichment broth	Method for carbapenem-R detection	N	n	Individual estimated preva-lence
							<1600 g + for all patients in NICU.	formic acid and/or alcohol for floors. No monitoring of colonization with resistant bacteria implemented.	cultures and antibiotics applied until negative result.)						
South Africa - (Ogunbosi, 2020)	yes	AFR	UM	no	cross-sectional study	H	Pediatric unit of a 273-bedded tertiary university hospital			Entero-bacteria	None	ChromID ESBL media + Vitek2 (CLSI) + MICs of ertapenem/meropenem/imipenem for confirmation	52	0	0.9% [0-8.2]
South Africa - (Russell, 2024)	yes	AFR	UM	yes, low birth weight	cross-sectional study nested in a RCT	H	Tertiary Hospital	according to the trial protocol (NeoCHG)		Entero-bacteria	None	culture on MacConkey agar + AST (disc diffusion, CLSI)	130	0	0.4% [0-3.4]
Sri Lanka - (Meredith, 2021)	yes	SEAR	LM	no	cohort study	NH			3/199 (1,5%)	Entero-bacteria	None	MacConkey agar with with 2 mg/L cefotaxime + AST and MIC measure with MicroScan (CLSI) + modified Hodge test + PCR for carbapenemase genes	199	0	0.2% [0-2.3]
Tanzania - (Marando, 2018)	yes	AFR	L	Yes, signs of sepsis	cohort study	H	Neonatal unit of a Tertiary University Hospital		82/304 (27%)	Entero-bacteria	None	MacConkey agar with 2 mg/L cefotaxime + disc diffusion (CLSI) + WGS	304	0	0.2% [0-1.5]
Thailand - (Roberts, 2019)	yes	SEAR	LM	no	cohort study	NH				Entero-bacteria	None	Bacteriological medium used unclear + disk diffusion (imipenem disk) (CLSI)	97	17	17.5% [11.1-26.4]
Turkey - (Akturk, 2016)	no_specific bacterial species	EUR	UM	no	cohort study	H	NICU from a tertiary university hospital	Active surveillance of CRKP and vancomycin-resistant enterococci (VRE) rectal colonization since 2013 (routine screening once a week)		Klebsiella pneu-moniae	None	Chromogenic agar with 1 mg/L meropenem + disk diffusion + MIC measure with E-test	1,671	44	

© 2024 Beaumont AL et al. *JAMA Network Open*.

Study ID	Included in m-a[1]	WHO region	WB cat.[2]	Spe-cial pop.	Design	Con text [3]	Hospital and ward structure	IPC measures [4]	ATB[5]	Bacteria	Pre-enrichme nt broth	Method for carbapenem-R detection	N	n	Individual estimated preva-lence
Turkey - (Orman, 2023)	yes	EUR	UM	Yes, refere d patient s	cohort study	H	NICU of a University Hospital, with 27 beds (25 beds in open area + 2 single rooms)	Doctor patient ratio: 1/7. Contact isolation was applied until screening results were obtained. If positive, patients were isolated, with cohorting of HCW.	125/ 125 (100%)	Entero-bacteria	yes, selective (Mueller-Hinton Broth with ertapenem)	EMB agar plate with ertapenem/meropenem disks	125	34	27.2% [20.1-35.6]
Vietnam - (Garpvall, 2021)	yes	WPR	LM	no	cohort study	NH				Entero-bacteria	None	ChromID Carba	323	151	46.7% [41.4-52.2]
Vietnam - (Tran, 2019)	yes	WPR	LM	no	cross-sectional study	NH				Entero-bacteria	None	ChromID Carba + Vitek2 AST (CLSI)	329	81	24.6% [20.3-29.6]
Vietnam - (Tran, 2019)	yes	WPR	LM	no	cross-sectional	H	Neonatal units from 12 hospitals from different regions of Vietnam			Entero-bacteria	None	ChromID Carba + Vitek2 AST (CLSI)	247	161	65.2% [59-70.9]

[1]m-a : included in meta-analysis of prevalence
[2]World Bank category, L : low-income country, LM : lower-middle income country, UM : upper-middle income country
[3]H : Hospitalised, N-H : Non-hospitalised
[4]IPC : infection prevention and control
5% of the study population receiving antibiotics, if extractible from the article
Empty grey cells indicate missing data (could not be extracted from the paper), or non-applicable information (i.e. hospital structure for community-based studies)

© 2024 Beaumont AL et al. *JAMA Network Open*.

eTable 4: Methods and results of studies reporting data on bacterial species or resistance genes.

A. Methods and results of studies reporting data on bacterial species of 3GC-R isolates

Study ID	N isolates	N *E. coli*	N *K. pneumoniae*	N *Enterobacter sp.*
Morocco - (Arhoune, 2021)	149	56	79	9
Gambia - (Bah, 2023)	4	3	1	-
Haiti - (Chaintarli, 2018)	29	14	10	0
Ethiopia - (Desta, 2016)	15	2	13	-
Madagascar - (Herindrainy, 2018)	55	17	14	0
Mexico - (Huerta-García, 2015)	102	8	77	17
Kenya - (Kagia, 2019)	65	25	31	8
India - (Kothari, 2013)	79	69	3	7
Ghana - (Labi, 2020)	132	25	90	0
Malaysia - (Lee, 2021)	39	3	36	0
Tanzania - (Marando, 2018)	173	60	112	5
Sri Lanka - (Meredith, 2021)	3	3	0	0
Serbia - (Milic, 2021)	13	7	5	0
Peru - (Nadimpalli, 2024)	81	47	34	0
Tanzania - (Nelson, 2014)	35	5	27	0
Ecuador - (Nordberg, 2013)	52	46	6	0
Nigeria - (Obadare, 2023)	105	12	36	31
South Africa - (Ogunbosi, 2020)	104	36	65	1
Thailand - (Roberts, 2019)	48	7	26	15
India - (Shakil, 2010)	139	88	51	0
Tanzania - (Silago, 2020)	83	14	49	4
Cambodia - (Turner, 2016)	218	97	121	0

B. Methods and results of studies reporting data on bacterial species of carbapenem-R isolates

Study ID	N isolates	N *E. coli*	N *K. pneumoniae*	N *Enterobacter sp.*
Morocco - (Arhoune, 2021)	49	26	18	2
India - (Kothari, 2013)	1	0	0	1
Ghana - (Labi, 2020)	18	0	18	0
Algeria - (Mairi, 2019)	7	0	7	0

© 2024 Beaumont AL et al. *JAMA Network Open*.

Serbia - (Mijac, 2023)	88	1	87	0
South Africa - (Ogunbosi, 2020)	1	0	0	1
Thailand - (Roberts, 2019)	17	0	17	0
India - (Singh, 2018)	26	2	18	2
Vietnam - (Tran, 2019)	199	89	99	11
Cambodia - (Turner, 2016)	1	1	0	0

C. **Methods and results of studies reporting data on resistance genes of 3GC-R isolates**

Study ID	Method	Sampling	Enzymes screened	N isolates	%CTXM	%CTXM-1	%SHV	%TEM	%AmpC
Morocco - (Arhoune, 2021)	PCR	all isolates	ESBL (blaCTX-M phylogenetic lineage groups 1, 2, and 9; bla TEM ; and bla SHV)	330		81%	70%	44%	
Kenya - (Edwards, 2023)	previously validated in-house high resolution melt (HRM) analysis qPCR assay	all samples	ESBL (CTX-M groups 1 and 9)	16	100%	88%	0%	0%	
Nigeria - (Edwards, 2023)	previously validated in-house high resolution melt (HRM) analysis qPCR assay	all samples	ESBL (CTX-M groups 1 and 9)	22	100%	100%			
Guinea-Bissau - (Isendahl, 2012)	multiplex, real-time TaqMan PCR assay	all confirmed ESBL isolates	ESBL (blaCTX-M genes into the four phylogenetic subgroups 1, 2, 9 and 8/25) + isolates negative for blaCTX-Mwere analyzed for carbapenemase, AmpC, ESBL blaTEMand blaSHVgenes with the commercial Check-MDR CT101 PCR assay	174	100%	97%			not done (all ESBL+)
India - (Kothari, 2013)	PCR	In 27 randomly selected neonates (22 strains)	ESBL (blaTEM, blaSHV, blaCTX-M[group1, 2, 8, 9 and 25]) and ampC (MOX , CIT , DHA , ACC , EBC ,and FOX)	22	64%		5%	5%	12/22 AmpC+ (5 DHA, 2 CMY-1, 3 CMY-2, 2 MOX, 1 FOX)
Malaysia - (Lee, 2021)	PCR	all isolates	ESBL (blaTEM,blaSHV,blaOXA-1, blaOXA-9,blaCTX-M-1,blaCTX-M-2 and blaCTX-M-9)	39	87%	85%	87%	54%	

© 2024 Beaumont AL et al. *JAMA Network Open*.

Tanzania - (Marando, 2018)	WGS+ResFinder	37 isolates	all R genes (ResFinder)	37	100%	100%			
Serbia - (Milic, 2021)	commercial Phusion U Green Multiplex PCR Master Mix (Thermo Fisher Scientific, Waltham, Massachusetts, USA) kit	41/55 ESBL-producing isolates	ESBL (blaTEM,blaSHV,blaCTX-M group 1 (CTX-M-15),blaCTX-M group 2, andblaCTX-M group 9)	41	54%		49%	63%	
Ecuador - (Nordberg, 2013)	probe-based PCR-assay	all isolates	ESBL (CTXM)	65	100%	98%			
Nigeria - (Obadare, 2023)	multiplex PCR	all confirmed ESBL-PE isolates	ESBL (blaSHV , blaTEM and blaCTX-M genes)	64	73%		44%	73%	
South Africa - (Ogunbosi, 2020)	PCR	94/104 isolates tested	ESBL (CTXM, SHV, TEM)	80	95%		14%	66%	
India - (Subhasree Roy , 2013)	PCR	all ESBL isolates	ESBL (blaTEM, blaSHV, blaOXA-1, and blaCTX-M genes)	162	100%	99%	0%	0%	

D. Methods and results of studies reporting data on resistance genes of carbapenem-R isolates

Study ID	Method	Sampling	Enzymes screened	(note)	N isolates	%OXA	%NDM	%KPC	%VIM
Morocco - (Arhoune, 2021)	PCR	all isolates	carbapenemase (OXA-48 , blaKPC , blaNDM , blaIMP, and blaVIM)		89	89%	0%	0%	0%
Kenya - (Edwards, 2023)	previously validated in-house high resolution melt (HRM) analysis qPCR assay	all samples	five main carbapenemase genes (blaVIM, blaIMP−1, blaKPC, blaNDM, blaOXA−48)		8	0%	88%	0%	13%
Nigeria - (Edwards, 2023)	previously validated in-house high resolution melt (HRM) analysis qPCR assay	all samples	five main carbapenemase genes (blaVIM, blaIMP−1, blaKPC, blaNDM, blaOXA−48)		21	5%	95%		0%
Ghana - (Labi, 2020)	WGS	all	all (ResFinder)	WGS si carbaR	18	100%	0%	0%	0%
Algeria - (Mairi, 2019)	PCR	all carbaR isolates	not described		28	100%	0%	0%	0%
Serbia - (Mijac, 2023)	multiplex PCR	all isolates	carbapenemase (blaKPC,blaNDM,blaIMP,blaVIM,blaOXA-48)		88	51%	1%	48%	0%

© 2024 Beaumont AL et al. *JAMA Network Open*.

South Africa - (Ogunbosi, 2020)	PCR	all carbaR isolates	carbapenemase (blaNDM, blaKP, blaOXA-48, blaIMP, blaVIM and blaGES)	*no gene detected*	*1*				*0%*
India - (Singh, 2018)	real-time PCR	all isolates	carbapenemase (NDM-1, IMP, VIM, and KPC). PCR for OXA-48 and -181 were not performed because of financial constraints	PCR for OXA-48 and -181 were not performed because of financial constraints	26		38%	0%	31%
China - (Wang, 2022)	PCR	all isolates	carbapenemase (blaKPC, blaNMC, blaGES, blaIMI and blaSME, blaVIM, blaIMP, blaNDM, blaSIM, blaSPM, blaGIM, blaOXA-48)		188	0%	100%	0%	0%

© 2024 Beaumont AL et al. *JAMA Network Open*.

eTable 5. Meta-regression multivariate models.

A. **Meta-regression model 1:** effect of selected study factors/covariates on the prevalence of C3G-R *Enterobacterales* colonization among neonates in LMICs.

Variable	Estimate	SE	OR	IC95%	p-value
Intercept	*25.2*	*90.1*	-	-	-
**Sampling context**					
Non-hospitalised individuals	Ref	Ref	**Ref**	Ref	-
Hospitalised patients	0.87	0.38	**2.4**	[1.1-5.2]	**0.03***
**Sampling time**					
Before 3 days of life	Ref	Ref	**Ref**	Ref	-
After 3 days of life	1.14	0.38	**3.1**	[1.4-6.8]	**<0.01***
**Study year**					
Study year (continuous)	-0.034	0.05	**0.99**	[0.9-1.08]	0.76

N = 30, one study was omitted because of missing data. Pseudo-R^2=52%.
SE: standard error. OR: odds ratio. IC95%: 95% confidence interval.
**p-values below 0.05 are considered statistically significant*

B. **Meta-regression model 2:** effect of selected study factors/covariates on the prevalence of carbapenem-R *Enterobacterales* colonization among neonates in LMICs.

Variable	Estimate	SE	OR	IC95%	p-value
Intercept	*-6.38*	*0.99*	-	-	-
**Sampling context**					
Non-hospitalised individuals	Ref	Ref	**Ref**	Ref	-
Hospitalised patients	1.75	0.92	**5.8**	[0.8-39.4]	0.07
**Region**					
Else than Pacific-Asia	Ref	Ref	**Ref**	Ref	-
Pacific Asia Region▪	2.71	0.93	**15.1**	[2.2-103.8]	**<0.01***
**Study year (data collection)**					
Before 2016 (included)	Ref	Ref	**Ref**	Ref	
After 2017▲	2.14	0.92	**8.5**	[1.3-57.3]	**0.03***

N = 25, one study was omitted because of missing data.Pseudo-R^2=57%.
SE: standard error. OR: odds ratio. IC95%: 95% confidence interval.
**p-values below 0.05 are considered statistically significant*
▪*Pacific Asia was defined as both Southeast and Western Pacific WHO regions.*
▲*2017 was the median year of data collection in included studies*

C. **Meta-regression model 3:** effect of selected study factors/covariates on the prevalence of MRSA colonization among neonates in LMICs.

Variable	Estimate	SE	OR	IC95%	p-value
Intercept	*-5.9*	*0.99*	-	-	-
**Hospitalisation in NICU**					
No	Ref	Ref	**Ref**	Ref	-
Yes	2.24	0.99	**9.4**	[1.05-84.5]	**0.046***
**Income category (World Bank)**					
Upper-middle income country	Ref	Ref	**Ref**	Ref	-
Low- and lower-middle income country	0.57	0.76	**1.8**	[0.33-9.43]	0.46
**Study year (data collection)**					
Before 2012 (included)	Ref	Ref	**Ref**	Ref	-
After 2013▲	2.24	0.99	**9.4**	[1.04-84.6]	**0.046***

N = 15, no study was omitted because of missing data.
SE: standard error. OR: odds ratio. IC95%: 95% confidence interval. Pseudo-R^2=34%.
**p-values below 0.05 are considered statistically significant*
▲*2013 was the median year of data collection in included studies*

© 2024 Beaumont AL et al. *JAMA Network Open*.

eTable 6: Description of risk factors investigated and main findings of studies reporting risk factors analysis for 3GC-R-E or CRE colonisation.

Study_id	Precise endpoint (from the text)	Investigated risk factors	Significant risk factors (multivariate analysis)
**Morocco - (Arhoune, 2021)**	colonisation with MDRE+ at NICU admission	Gender, age, prematurity, birth weight, comorbidities, birthplace, admission route, delivery mode, venous catheterization, breastfeeding, antibiotherapy	Admission route (ref = house, newborns admitted from maternity of university hospital (aOR 3.1 [1.9–5.1]), newborns admitted from other wards (aOR 2.0 [1.3–3.23])); neurological distress (aOR 2.2 [1.0–4.7])
**Malaysia - (Boo, 2005)**	colonisation with KP-BLSE at NICU	Birth weight, gestational age, age of admission, congenital malformation, respiratory distress syndrome, mechanical ventilation, nasal CPAP therapy, use of glycerine suppositories, umbilical arterial catheterization, arterial line catheterization, urinary catheterization, surgery, TPN, transfusion with blood components, umbilical venous catheterization, given cephalosporin or imipenem therapy	Duration of hospital stay (aOR 1.3 [1.2-1.4]), early-onset pneumonia (aOR 8.3 [1.6-43.4])
**Kenya - (Kagia, 2019)**	colonisation with ESBL-PE in neonates admitted to hospital	Sex, age, weight at admission, prematurity, place of delivery, mothers' age, main water source, breastfeeding, type of toilet, number of people living in the same house	Age at admission (ref = day of birth, aOR 1.7 [0.7-4.3] for 1-2 days, aOR 3.9 [1.5-10.2] for 3-28 days)
**Ghana - (Labi, 2020)**	colonisation with C3G-R bacteria in neonates in NICU	Weight at admission, duration of stay before sample collection, mother's age, parity, sex, type of delivery, prematurity, prolonged rupture of membranes	Duration of stay before specimen collection in days (aOR 1.1 [1.05-1.14]), antibiotic use (aOR 15 [7.9-28.6])
**China – (Ma, 2014)**	Carbapenemase-producing Klebsiella pneumoniae colonization in neonates hospitalized in the neonatal intensive care unit (NICU)	gestational age, birth weight, length of hospital stay, duration of mechanical ventilation, congenital heart disease, peripherally inserted central catheter, surgical operation, duration of intravenous nutrition, carbapenems use, duration of carbapenems use and glycopeptides use	exposure to more than 4 days of carbapenems use (aOR 18.7 [1.98-175.5])
**Algeria - (Mairi, 2019)**	acquisition of carbapenemase-producing Enterobacteriaceae in newborns from a maternity	Mother's age, maternal antibiotic use in the past 3 months, previous hospital admission, chronic disease, surgical intervention, sex, mode of delivery, low birth weight	Low birth weight (aOR not reported, p < .01)
**Tanzania - (Marando, 2018)**	ESBL-PE colonisation among inpatients neonates with signs of sepsis	Age at admission, sex, admission route, birth weight, body temperature, oxygen sat, skin pustule, umbilical discharge, history of antibiotic for the baby, type of ward, maternal fever, prolonged rupture of membrane, maternal antibiotics	History of antibiotic - baby (aOR 1.7 [1-2.9]), maternal colonization with ESBL (aOR 2.2 [1.3-3.8])

© 2024 Beaumont AL et al. *JAMA Network Open*.

**Sri Lanka - (Meredith, 2021)**	colonisation with MDRE+ in newborns from a maternity	Mother's age, infant gender, low-birth weight, breastfeeding, average monthly household income, mother's medical history, number of adults/children at home, complications during pregnancy, mode of delivery, difficulties with delivery, infant hospitalization, antibiotic use, housing details, toilet type, household water treatment methods, mothers colonization	Mother's colonization at enrollment (aOR = 3.6 [1.0–12.6]), mother's colonization at reassessment (aOR = 4.4 [1.4–14.3]), delivery by C-section (aOR = 2.91 [1.0–8.5]), and low birth weight (aOR = 5.4 [1.4–20.3])
**Serbia - (Mijac, 2023)**	colonisation with CRE during neonatal unit stay	Sex, prematurity, one-minute apgar score, birth weight <1000g, small for gestational age, invasive procedures, complications of prematurity, sepsis, stay at NICU, days of hospitalization	No multivariate analysis was conducted
**Ecuador - (Nordberg, 2013)**	colonisation with ESBL-producing gram-negative bacteria in a NICU	Sex, gestational age, birth weight, length of NICU stay, invasive procedures, feeding mode, antibiotic use and treatment duration, 5-min Apgar score, malformations	Length of stay in NICU 21–30 days (aOR 15.8 [2.6–97.5]) and 30 days (aOR 71.8 [6.1–848.4]), enteral feeding with a combination of breastfeeding and formula feeding (aOR 9.5 [1.9–46.8])
**Nigeria - (Obadare, 2023)**	ESBL-PE neonatal rectal colonisation in the neonatal ward	Sex, age at admission, place of delivery, mode of delivery, maternal antibiotic use, PROM, number of babies in the same room, known ESBL-PE carrier in the room, environmental conditions, medical diagnosis	PROM, number of neonates clustered in a room, and the presence of an ESBL-PE coloniser in the same room (aOR not reported)
**Thailand - (Roberts, 2019)**	colonisation with ESBL-producing Ecoli or KP at NICU admission	Prematurity, birth asphyxia, sex, ventilation-endotracheal tube	Male sex (aOR 3.23 [1.2-8.6])
**Brésil - (Sakai, 2020)**	colonisation with MDR micro-organism at discharge from the neonatal unit	Delivery mode, gestational age, corrected age at discharge, birth weight, discharge weight, maternal breastfeeding at discharge, length of hospitalization, invasive procedures	Duration of antibiotic treatment ≥ 15 days (aOR = 2.18 [1.4-3.5])
**India - (Shakil, 2010)**	acquisition of ESBL-producing Ecoli or Kp in a NICU	Birthweight, gestational age, sex, mode of delivery, parity, maternal medical history, meconium-stained liquid, PROM, medical complications of prematurity, various biological parameters, Apgar score, length of stay in NICU, medications, invasive procedures, educational status of mother	Length of stay in the NICU (aOR not reported)
**India - (Singh, 2018)**	CRE gut colonisation over the all study (acquisition during the 10st days of life of NICU neonates)	Birth weight, PROM, mode of delivery, prematurity, duration of hospital stay, feeding mode, ventilation, antibiotic administration	top feeding (aOR 6.41[2.53-16.28]), antibiotics to neonate (aOR 7.10 [2.76-18.24])
**India - (Subhasree Roy , 2013)**	colonisation with ESBL-producing Ecoli in hospitalized neonates	birth location, birth weight, gestational age, mode of delivery, maternal risk factors, ventilation, NICU stay, antibiotic administration	NICU stay (aOR 3.7 [1.1-11.8])

© 2024 Beaumont AL et al. *JAMA Network Open*.

**Cambodia - (Turner, 2016)**	colonisation with an C3G-R micro-organism on admission to a neonatal unit	prematurity, PROM, birth location, severe medical situation (ventilation, CPAP or inotropes), admission to another department before	Delivery in hospital versus home/health center (aOR 3.0 [1.7-5.4]), PROM (aOR 3.8 [1.0-25.0]), severe (aOR 2.1 [1.0-5.0])
**China - (Wang, 2022)**	colonisation with carba-R Enterobacterales versus carba-S enterobacterales in the first 3 days of life	Age at admission, gender, gestational age, birth weight, prematurity, cesarean section, medical conditions, type of antibiotics used, oxygen, invasive procedures, type of feeding, residence location	C-section (aOR 2.9 [1.7-5.1]), acidosis (aOR 2.3 [1.2-4.3]), respiration failure (aOR 3.5 [1.4-8.5]), gastric lavage (aOR 3.1 [1.5-6,4]), enema (aOR 2.8 [1.7-4.9])
**Ethiopia - (Zakir, 2021)**	colonisation with ESBL-producing gram-negative bacteria among hospitalized neonates	Sex, gestational age, birth weight, age at admission, birth place, mode of delivery, feeding type, reason for hospitalization, exposure to invasive device, antibiotic treatment, NICU length of stay	Endotracheal intubation (aOR 4.2 [1.8–9.5]), treatment with ampicillin+gentamicin (aOR 3.3[1.5–7.6]), staying in the NICU between 11–20 days (aOR = 2 [1.0–4.5])

© 2024 Beaumont AL et al. *JAMA Network Open*.

eTable 7: Description of studies included for MRSA prevalence.

Study ID	Included in m-a[1]	WHO region	WB cat.[2]	Special pop.	Design	Context[3]	Hospital and ward structure	IPC measures[4]	ATB[5]	Sampling type	Enrichment	Culture Medium	MRSA id.	N	n	Individual estimated prevalence
Ghana - (Walana, 2020)	yes	AFR	LM	no	cross-sectional	H	Teaching referral hospital			nasal swab	None	Mannitol salt agar plate	resistance to oxacillin by disk diffusion method (CLSI guidelines)	19	4	21.1% [8-43.9]
Brazil - (Silva, 2003)	yes	AMR	UM	no	cross-sectional	H	Tertiary university hospital, neonatal ward of 45 beds		33%	nasal + mouth + nostril swab	yes, non-selective (Trypticase Soy Broth)	Mannitol salt agar plate	cultivation on agar containing NaCl 4% and 6 mg/L oxacillin	143	30	21% [15.1-28.4]
Brazil - (Vieira, 2014)	yes	AMR	UM	no	cohort study	H	Pediatric healthcare centers, 4 NICUs with 69 beds in total			nasal swab	None	Mannitol salt agar plate	resistance to cefoxitin and oxacillin by disk diffusion method (CLSI guidelines)	701	4	0.6% [0.2-1.5]
Gabon - (Schaumburg, 2013)	yes	AFR	UM	no	cohort study, nested in an RCT	N-H			100%	nasal + pharyngeal	None	Blood agar plate with one aztreonam disk	Resistance to oxacilin by Vitek 2 (EUCAST guideline) and SCCmec typing	318	0	0.2% [0-1.4]
China - (Geng, 2020)	yes	WPR	UM	no	cross-sectional	N-H			37%	nasal swab	None	Blood agar plate	resistance to cefoxitin by disk diffusion method (CLSI guidelines) and mecA gene detection	516	28	5.4% [3.8-7.8]
China - (Lin, 2018)	yes	WPR	UM	no	cohort study	N-H				nares + mouth + skin (axilla, around the eyes, periumbilical area, groin)	yes, non-selective (tryptone, sodium chloride, mannitol, yeast extract)	Mannitol salt agar plate	resistance to cefoxitin by disk diffusion method (CLSI guidelines) and SCCmec typing	1,834	15	0.8% [0.5-1.4]
China - (Ai, 2020)	no	WPR	UM	no	cross-sectional	H	Three medical centers in different			feces	None	Mannitol salt agar plate	Resistance to oxacilin by Vitek 2	446	20	

© 2024 Beaumont AL et al. *JAMA Network Open*.

Study ID	Included in m-a[1]	WHO region	WB cat.[2]	Special pop.	Design	Context[3]	Hospital and ward structure	IPC measures[4]	ATB[5]	Sampling type	Enrichment	Culture Medium	MRSA id.	N	n	Individual estimated prevalence
							areas of Guangzhou (no more information)						(CLSI guidelines) + PCR (mecA)			
Benin - (Ahoyo, 2006)	yes	AFR	L	no	cross-sectional	H	Departmental public hospital, neonatology ward with 40 beds divided in 3: NICU, pre-term, conventional	Environmental samples were also made during the study		nasal swab	None	Mannitol salt agar plate	resistance to oxacillin by disk diffusion method using 5% NaCl Mueller Hinton agar plate (CA-SFM guidelines)	290	5	1.7% [0.6-4.1]
Brazil - (Garcia, 2014)	yes	AMR	UM	no	cohort	H	Tertiary public university hospital, neonatal unit (65 beds). 5 sectors: NICU (8), high-risk stepdown (9), medium-risk (15), well baby nursery (25), private sector (8).	Hand hygiene: alcohol hand rubs, hand washing: plain soap +chlorhexidine. Most babies are roomed with their mothers. HCW Colonization also evaluated in the study.	12%	anterior nares + oro-pharynx + perineum + umbilical stump	None	Blood agar plate	cultivation on Mueller Hinton agar plate with NaCl 4% and 6 mg/L oxacillin and mecA gene detection	403	53	13.2% [10.2-16.8]
Morocco - (Mourabit, 2017)	yes	AFR	LM	no	cross-sectional	N-H			0% (exclusion criteria)	nasal swab (both anterior nares)	yes, non-selective (brain-heart infusion)	Mannitol salt agar plate	resistance to cefoxitin by disk diffusion method (CLSI guidelines) and mecA gene detection	312	3	1% [0.2-2.9]
Cape Verde - (Aires-De-Sousa, 2015)	yes	AFR	LM	no	cross-sectional	H	Two public general hospitals			nasal swab	None	Selective chromogenic medium	resistance to cefoxitin by disk diffusion method (CLSI guidelines) and mecA gene detection	33	1	3% [0-16.7]

© 2024 Beaumont AL et al. *JAMA Network Open*.

Study ID	Inclu-ded in m-a[1]	WHO re-gion	WB cat.[2]	Special pop.	Design	Context[3]	Hospital and ward structure	IPC measures[4]	ATB[5]	Sam-pling type	Enrich-ment	Culture Medium	MRSA id.	N	n	Individual estimated prevalence
Brazil - (Salgueiro, 2019)	yes	AMR	UM	no	cross-sectional	H	Public hospital, NICU with 20 beds (5 ICU+15 intermediate unit)			nasal (or periumbilical if no access to nasal site)	None	Mannitol salt agar plate	mecA gene detection	175	2	1.1% [0-4.3]
Pakistan - (Malik, 2023)	yes	SEAR	LM	no	cross-sectional	N-H				nasal swabs	None	Blood agar plate	R to methicillin on AST	80	16	20% [12.6-30.1]
South Africa - (Mabena, 2024)	yes	AFR	UM	no	cohort study	N-H				nasal (anterior nares) + skin swab (umbilicus, axilla, groin)	yes, non-selective (brain heart infusion)	Chromogenic Orientation Agar plates	unclear	102	8	7.8% [3.8-14.9]
South Africa - (Russell, 2024)	yes	AFR	UM	yes_low birth weight	cross-sectional study nested in RCT	H	Tertiary Hospital	according to the trial protocol (NeoCHG)		nose + cervical skin folds & umbilicus	None	Blood agar plate	Kirby bauer disc diffusion AST (R to cefoxitin)	130	0	0.4% [0-3.4]
Ban-gladesh - (Russell, 2024)	yes	SEAR	LM	yes_low birth weight	cross-sectional study nested in RCT	H	Children's hospital	according to the trial protocol (NeoCHG)		nose + cervical skin folds & umbilicus	None	Blood agar plate	Kirby bauer disc diffusion AST (R to cefoxitin)	78	3	3.8% [0.9-11.2]

[1]m-a : meta-analysis of prevalence
[2]World Bank category, L : low-income country, LM : lower-middle income country, UM : upper-middle income country
[3]H : Hospitalised, N-H : Non-hospitalised
[4]IPC : infection prevention and control
[5]% of the study population receiving antibiotics, if extractible from the article
Empty grey cells indicate missing data (could not be extracted from the paper).or non-applicable information (i.e. hospital structure for community-based studies)

© 2024 Beaumont AL et al. *JAMA Network Open*.

eTable 8: Description of risk factors investigated and main findings of studies reporting risk factors analysis for MRSA colonisation.

Study_id	Precise endpoint (from the text)	Investigated risk factors	Significant risk factors (multivariate analysis)
**Brazil - (Garcia, 2014)**	Risk factors for MRSA carriage by newborns	Gender, twinning, birth weight, gestational age at birth, Apgar, breastfeeding, capillary blood glucose check, vaginal delivery, presence of comorbidities, use of several procedures (ventilation, venous catheter, parenteral nutrition, surgery, dialysis, drains), use of antimicrobial drugs, maternal comorbidities, maternal tobacco/alcohol/drug use, maternal education, maternal skin disorder, maternal history of surgery, skin disorders among family members, maternal hospitalisation for > 1 month before delivery, discharge at day 3 of life, blood transfusion, maternal rhinosinusitis, amniotic liquid not clear, prolonged rupture of membranes, family > 3 members, MRSA-colonised mother	Among the entire population of newborns : mother with < 4 years of formal education (aOR 2.99 [1.10-8.07]) and maternal rhinosinusitis (aOR 0.33 [0.12-0.88])<br><br>Among newborns hospitalised > 72h : breastfeeding (aOR 0.17 [0.04-0.80])
**Brazil - (Silva, 2003)**	Risk factors for acquiring MRSA	Sex, birth weight, Apgar, gestational age, antibiotic use, length of hospitalization, incubator care, mechanical ventilation, invasive devices	No multivariate analysis was conducted

© 2024 Beaumont AL et al. *JAMA Network Open*.

eReferences

1. Bezabih, Y. M. *et al.* The global prevalence and trend of human intestinal carriage of ESBL-producing *Escherichia coli* in the community. *J. Antimicrob. Chemother.* **76**, 22–29 (2021).
2. Nourollahpour Shiadeh, M. *et al.* Worldwide prevalence of maternal methicillin-resistant Staphylococcus aureus colonization: A systematic review and meta-analysis. *Microb. Pathog.* **171**, 105743 (2022).
3. Tesfa, T., Mitiku, H., Edae, M. & Assefa, N. Prevalence and incidence of carbapenem-resistant K. pneumoniae colonization: systematic review and meta-analysis. *Syst. Rev.* **11**, 240 (2022).
4. Harrer, M., Cuijpers, P., A, F. T. & Ebert, D. D. *Doing Meta-Analysis With R: A Hands-On Guide*. (Chapman & Hall/CRC Press, Boca Raton, FL and London, 2021).
5. Magiorakos, A.-P. *et al.* Multidrug-resistant, extensively drug-resistant and pandrug-resistant bacteria: an international expert proposal for interim standard definitions for acquired resistance. *Clin. Microbiol. Infect.* **18**, 268–281 (2012).

© 2024 Beaumont AL et al. *JAMA Network Open*.
