# Supplementary material for: Neonatal Colonization With Antibiotic-Resistant Pathogens in Low- and Middle-Income Countries: A Systematic Review and Meta-Analysis
Source: JAMA Netw Open. 2024 Nov 5;7(11):e2441596. doi: 10.1001/jamanetworkopen.2024.41596 (PMC11581591; doi:10.1001/jamanetworkopen.2024.41596)
Supplement: Supplement 2. — Data Sharing Statement [file jamanetwopen-e2441596-s002.pdf]

Data Sharing Statement

Beaumont. Neonatal Colonization With Antibiotic-Resistant Pathogens in Low- and Middle-Income Countries. *JAMA Netw Open*. Published November 05, 2024. doi:10.1001/jamanetworkopen.2024.41596

Data
Data available: Yes
Data types: Data (not involving human participants), Data dictionary
How to access data: Dataset and data dictionary will be available at publication at the following link (not activated at submission time) :
https://entrepot.recherche.data.gouv.fr/dataset.xhtml?persistentId=doi:10.57745/MIIONK
When available: With publication

Supporting Documents
Document types: None

Additional Information
Who can access the data: Dataset will be freely available to anyone requesting the data.
Types of analyses: Dataset will be freely available, for any scientific purpose.
Mechanisms of data availability: The dataset will be available for open access download.
